# Cost-effectiveness analysis of nivolumab combination therapy in the first-line treatment for advanced esophageal squamous-cell carcinoma

**DOI:** 10.3389/fonc.2022.899966

**Published:** 2022-07-22

**Authors:** Shixian Liu, Lei Dou, Kaixuan Wang, Zhao Shi, Ruixue Wang, Xiaohong Zhu, Zehua Song, Shunping Li

**Affiliations:** ^1^ Centre for Health Management and Policy Research, School of Public Health, Cheeloo College of Medicine, Shandong University, Jinan, China; ^2^ National Health Commission (NHC) Key Laboratory of Health Economics and Policy Research, Shandong University, Jinan, China; ^3^ Center for Health Preference Research, Shandong University, Jinan, China

**Keywords:** nivolumab, ipilimumab, chemotherapy, cost-effectiveness, esophageal squamous-cell carcinoma, first-line treatment

## Abstract

**Objective:**

We aimed to investigate the cost-effectiveness of nivolumab plus chemotherapy and nivolumab plus ipilimumab versus chemotherapy in the first-line treatment for advanced esophageal squamous-cell carcinoma (ESCC) patients from a healthcare system perspective in China.

**Methods:**

On the basis of the CheckMate 648 trial, a partitioned survival model was constructed to estimate economic costs and health outcomes among overall and PD-L1-positive advanced ESCC patients over a 10-year lifetime horizon. The health-related costs and utilities were obtained from the local charges and published literature. The lifetime costs, life-years, quality-adjusted life-years (QALYs), and incremental cost-effectiveness ratio (ICER) were measured. One-way and probabilistic sensitivity analyses (PSA) were performed to assess the robustness of the model.

**Results:**

In the base-case analysis, in overall and PD-L1-positive advanced ESCC patients, the ICERs were $415,163.81/QALY and $216,628.00/QALY for nivolumab plus chemotherapy, and$430,704.11/QALY and $185,483.94/QALY for nivolumab plus ipilimumab, respectively, compared with chemotherapy. One-way sensitivity analyses revealed that patients’ weight was the most influential parameter on ICER. The PSA demonstrated that the probability of nivolumab combination therapy being cost-effective was 0% over chemotherapy at the current price and willingness-to-pay threshold ($38,351.20/QALY). When the price of nivolumab and ipilimumab decreased 80%, the cost-effective probability of nivolumab plus ipilimumab increased to 40.44% and 86.38% in overall and PD-L1-positive advanced ESCC patients, respectively.

**Conclusion:**

Nivolumab combination therapy could improve survival time and health benefits over chemotherapy for advanced ESCC patients, but it is unlikely to be a cost-effective treatment option in China.

## Introduction

Esophageal cancer ranks seventh in terms of incidence (604, 000 new cases) and sixth in mortality (544, 000 deaths) worldwide, and East Asian countries were with the highest incidence rates, in part because of the enormous burden in China (1). Nearly half of the esophageal cancer across the world were in China, and the prevention of esophageal cancer has become an important goal for the Chinese government (2). Esophageal squamous-cell carcinoma (ESCC) and esophageal adenocarcinoma are the two major histological types of esophageal cancer, the former accounts for approximately 85% of the cases (3). Standard platinum plus fluorouracil or paclitaxel-based chemotherapy are the recommended first-line treatment option for patients with unresectable advanced, recurrent or metastatic ESCC (4, 5). Although chemotherapy has been widely used as first-line treatment for decades, survival improvement in these patients remains poor (median survival, <1 year) (6, 7), and novel treatment strategies are urgently needed.

Nivolumab, a human monoclonal anti-PD-1 antibody, has been demonstrated to improve the survival benefits for the treatment of several solid tumors in previously published studies (8–10). Programmed death ligand 1 (PD-L1) expression is enriched in ESCC, with expression ranging from 15% to 83% in tumor cells, and from 13% to 31% in immune cells (11). Recently, the results of CheckMate 648 trial, which compared nivolumab plus chemotherapy, nivolumab plus the monoclonal antibody ipilimumab, and chemotherapy in patients with unresectable advanced, recurrent, or metastatic ESCC, have revealed that overall survival (OS) was significantly longer with nivolumab plus chemotherapy than with chemotherapy alone in the overall population (median, 13.2 vs. 10.7 months; hazard ratio [HR], 0.74; 99.1% confidence interval [CI], 0.58-0.96; P=0.002) and also among patients with tumor-cell PD-L1 expression of 1% or greater (median, 15.4 vs. 9.1 months, HR, 0.54; 99.5% CI, 0.37-0.80; P<0.001) (12). A significant OS benefit was also seen with nivolumab plus ipilimumab over chemotherapy alone in overall and PD-L1-positive advanced ESCC patients (12). The CheckMate 648 trial indicated that nivolumab combination therapy could be considered as novel standard first-line treatment options to clinicians and decision-makers for the treatment of advanced ESCC patients, and these treatments has been recommended by the Chinese Society of Clinical Oncology (CSCO) Guidelines of Esophageal Cancer (13).

Significant costs always accompany the research and development of innovative drugs (14). The high cost of nivolumab and ipilimumab may limit its availability and impose a substantial financial burden on the national healthcare system. Although previous economic evidence demonstrated that nivolumab was unlikely to be cost-effective compared with chemotherapy in the second-line treatment of advanced ESCC patients from the perspective of Chinese society (15), the cost-effectiveness of nivolumab plus chemotherapy and nivolumab plus ipilimumab was not clear yet. Therefore, the objective of this study was to assess the cost-effectiveness of nivolumab combination therapy as first-line management for advanced ESCC patients in China. Such evidence may better inform clinical practice and reimbursement policy to optimize resource utilization.

## Methods

### Patients and intervention

This economic evaluation study was based on the CheckMate 648 trial (12), and the ethical approval of the institutional review board was exempted because no real human participants were involved. This study followed the Consolidated Health Economic Evaluation Reporting Standards 2022 (CHEERS 2022) reporting guidelines (**Supplementary Table 1**) (16). The target patient population was kept with the cohort included in the CheckMate 648 trial, an open-label, phase 3 trial conducted at 182 sites in 26 countries. Eligible patients were at least 18 years of age and had been confirmed unresectable advanced, recurrent, or metastatic ESCC, regardless of PD-L1 expression status, according to Response Evaluation Criteria in Solid Tumors (12).

Included patients were randomly assigned in a 1:1:1 ratio to receive nivolumab (240 mg intravenously on day 1 and day 15 every 4 weeks) plus chemotherapy (consisting of fluorouracil at a dose of 800 mg per square meter of the body-surface area on days 1 through 5 and cisplatin at a dose of 80 mg per square meter on day 1 each 4-week); nivolumab (3 mg per kilogram of body weight every 2 weeks) plus ipilimumab (1 mg per kilogram every 6 weeks); or chemotherapy alone until disease progression, unacceptable toxicity or other reasons (12). Patients were permitted to receive nivolumab or nivolumab plus ipilimumab up to a maximum of 2 years in line with package insert information and published resource. Subsequently, patients were managed with chemotherapy until progression.

### Model structure

**Figure 1:**
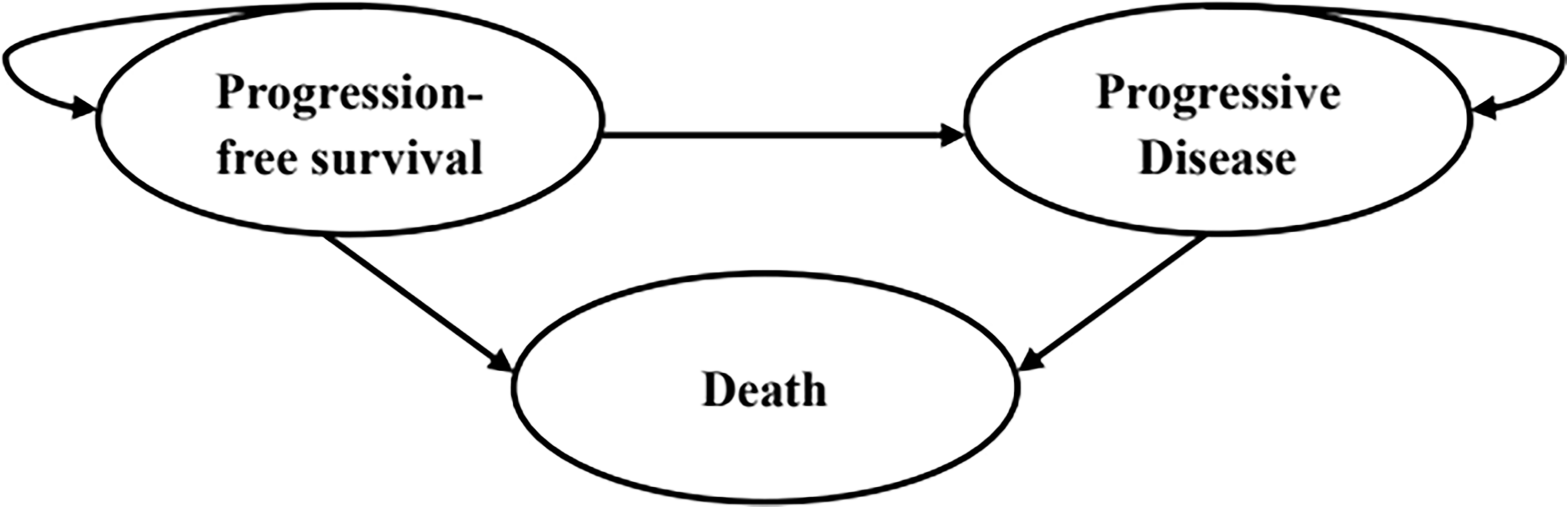
The structure of the partitioned survival model.

A partitioned survival model was developed using Microsoft Excel 2019 to compare the cost and effectiveness of the three competing regimens mentioned above among patients with advanced ESCC. The model was composed of three mutually exclusive health states: progression-free survival (PFS), progressed disease (PD) and death (**Figure 1**). The initial health state of all patients was PFS state, and that they could maintain their assigned health state or redistribute to another health state during each cycle. The proportion of patients in the PFS state at each time point was estimated as the area under the curve (AUC) for the PFS, while the proportion of patients in the death state was calculated by 1 minus the OS curve. The AUC between the PFS and OS curves was the PD state. The cycle length of the model was set at 4 weeks to facilitate parameter calculation. The time horizon was ten years to ensure that ESCC patients fully entered the terminal state.

This study was conducted from a Chinese healthcare system perspective. The primary outcomes of the model were total cost, life-years, quality-adjusted life-years (QALYs), and incremental cost-effectiveness ratio (ICER) between the treatment strategies. ICER was described as the additional cost required for each additional QALY. A half-cycle correction was implemented to improve the accuracy of the results. According to China Guidelines for Pharmacoeconomic Evaluations, a 5% annual discount rate was applied for all costs and QALYs (17). Based on the local Consumer Price Index, all costs were adjusted to 2022 prices and converted into US dollars (1$=6.33 CNY). As recommended by the World Health Organization, we used three times of the per capita gross domestic product (GDP) of China in 2021 ($38,351.20) as the willingness-to-pay (WTP) threshold to determine the cost-effectiveness of treatment regimens (18–21). Treatment options were considered highly cost-effective when the ICER was less than 1 times GDP per capita, while treatment options were considered cost-effective when the ICER was less than 3 times the GDP per capita (18). This WTP threshold has been widely employed in health technology assessment within low- and middle-income countries (20).

### Clinical data

The clinical efficacy and safety data were derived from the CheckMate 648 trial (12). As individual patient data (IPD) was not available, the GetData Graph Digitizer 2.26 (http://www.getdata-graph-digitizer.com/) was used to extract PFS and OS data points from the corresponding Kaplan–Meier survival curves. Different parametric distributions, including Exponential, Weibull, Log-logistic, Log-normal, and Gompertz, were fitted to extrapolate the survival curves beyond the follow-up duration of the clinical trials (22). The distribution with the best fit was evaluated based on graphical validation, Akaike information criterion (AIC) and Bayesian information criterion (BIC) (**Supplementary Tables 2, 3**) (23). The AIC and BIC were calculated using survival analyses with Stata 15.1. As for the long-tail curve, we used the sub-optimal or Weibull distribution for extrapolation to avoid overestimating the survival time (24). A total of 12 parametric survival curves were modeled, including the PFS and OS of overall and PD-L1-positive advanced ESCC patients (**Supplementary Figures 1–12**). The estimated scale (λ) and shape (γ) parameters of the fitting model are presented in **Table 1**.

**Table 1:** Optimal distribution of progression-free and overall survival curves.

Group		Shape	Scale	Distribution
**Overall advanced ESCC patients**				
Chemotherapy	PFS	1.670192	-0.081726	Log-normal
	OS	2.450230	-0.620517	Log-logistic
Nivolumab plus chemotherapy	PFS	1.908558	-0.514040	Log-logistic
	OS	2.659128	-0.598290	Log-logistic
Nivolumab plus ipilimumab	PFS	1.364702	-0.329654	Log-logistic
	OS	2.604049	-0.305029	Log-logistic
**Advanced ESCC patients with PD-L1-positive status**				
Chemotherapy	PFS	1.539710	-0.162895	Log-normal
	OS	2.431702	-0.627434	Log-logistic
Nivolumab plus chemotherapy	PFS	1.996054	0.103059	Log-normal
	OS	2.845191	0.070365	Log-normal
Nivolumab plus ipilimumab	PFS	1.657211	0.379699	Log-normal
	OS	2.809128	0.381912	Log-normal

ESCC, esophageal squamous-cell carcinoma; PFS, progression-free survival; OS, overall survival.

### Costs

Only direct medical costs were considered, including costs for drugs, laboratory tests and radiological examinations, routine follow-up, management of treatment-related severe adverse events (AEs), salvage therapy, best supportive care, and terminal care in end-of-life. The drug administration schedules were in accordance with the CheckMate 648 trial. To estimate the dosage of chemotherapy agents, a typical patient weighed 65 kg and had a height of 1.64 m was assumed, resulting in a body surface area of 1.72 m^2^ (25). The model included management costs associated with grade 3-4 AEs that occurred in 3% or greater of patients as they have a substantial effect on the survival and costs. In this condition, our analysis calculated the costs of nausea, decreased appetite, stomatitis, anemia, neutropenia, fatigue, and vomiting. The treatment of neutropenia covered that of leukopenia, so that the cost of leukopenia was not included based on expert consensus (26). Furthermore, owing to the unavailability of cost and disutility values, mucosal inflammation was not considered either. All costs were acquired from local hospitals or previously published literature (27–30). The nivolumab patient assistance program (PAP) was currently implemented in patients with advanced or recurrent gastric or gastro-oesophageal junction adenocarcinoma, so we only considered the effection of price reductions for nivolumab and ipilimumab.

### Utilities

Each health state was assigned a utility value anchored in 0 (death) and 1 (perfect health) in this partitioned survival model. QALYs were measured to determine health outcomes, namely, the utility values in a particular health state multiplied by the years of the corresponding state lasted. As the CheckMate 648 trial did not report the utility values of different health states, we obtained from another published study, a global, randomized, double-blind phase III trial, in which the utility values were measured by the EuroQol five dimensions health status questionnaire (EQ-5D-3L) and the UK-specific value algorithm (31, 32). In addition, we considered the disutility values caused by grade 3-4 AEs according to the relevant literature (33–35). All costs and utilities are shown in **Table 2**.

**Table 2:** Basic parameters input to the model and the ranges of the sensitivity analysis.

Parameters	Baseline value	Range		Distribution	Reference
		Minimum	Maximum		
**Cost inputs (US $)**					
Nivolumab (40 mg)	724.11	579.29	868.93	Gamma	Local estimate
Fluorouracil (250 mg)	31.42	25.13	37.70	Gamma	Local estimate
Cisplatin (10 mg)	1.47	1.18	1.77	Gamma	Local estimate
Ipilimumab (50 mg)	4,420.38	3,536.30	5,304.45	Gamma	Local estimate
Laboratory tests and radiological examinations	357.34	285.87	428.81	Gamma	(27)
Routine follow-up per cycle	73.72	58.98	88.47	Gamma	(27)
Salvage therapy	639.75	511.80	767.70	Gamma	(27)
Beat supportive care per cycle	182.23	145.78	218.68	Gamma	(27)
Terminal care in end-of-life	1,460.30	1,055.30	2,085.70	Gamma	(28)
Nausea per event	71.00	56.80	85.20	Gamma	(29)
Decreased appetite per event	115.00	92.00	138.00	Gamma	(29)
Stomatitis per event	46.54	37.23	55.85	Gamma	(30)
Anemia per event	523.36	418.69	628.03	Gamma	(30)
Decreased neutrophil count per event	454.26	363.41	545.11	Gamma	(30)
Fatigue per event	113.59	90.87	136.31	Gamma	(30)
Vomiting per event	71.00	56.80	85.20	Gamma	(29)
**Utility inputs**					
Progression-free survival	0.75	0.60	0.90	Beta	(31, 32)
Progressive Disease	0.60	0.48	0.72	Beta	(31, 32)
**Disutility inputs**					
Nausea	-0.13	-0.10	-0.15	Beta	(33)
Decreased appetite	-0.07	-0.05	-0.08	Beta	Assumption
Stomatitis	-0.15	-0.12	-0.18	Beta	(34)
Anemia	-0.07	-0.06	-0.09	Beta	(35)
Decreased neutrophil count	-0.20	-0.16	-0.24	Beta	(33)
Fatigue	-0.07	-0.05	-0.08	Beta	(33)
Vomiting	-0.13	-0.10	-0.16	Beta	(33)
**Risk of severe adverse events in chemotherapy group**					
Nausea	3.00%	2.40%	3.60%	Beta	(12)
Decreased appetite	3.00%	2.40%	3.60%	Beta	(12)
Anemia	6.00%	4.80%	7.20%	Beta	(12)
Decreased neutrophil count	8.00%	6.40%	9.60%	Beta	(12)
Fatigue	4.00%	3.20%	4.80%	Beta	(12)
Vomiting	3.00%	2.40%	3.60%	Beta	(12)
**Risk of severe adverse events in Nivolumab plus chemotherapy group**					
Nausea	4.00%	3.20%	4.80%	Beta	(12)
Decreased appetite	4.00%	3.20%	4.80%	Beta	(12)
Stomatitis	6.00%	4.80%	7.20%	Beta	(12)
Anemia	10.00%	8.00%	12.00%	Beta	(12)
Decreased neutrophil count	8.00%	6.40%	9.60%	Beta	(12)
**Proportion of patients receivied subsequent therapy**					
Chemotherapy	59.57%	47.65%	71.48%	Beta	(12)
Nivolumab plus chemotherapy	57.32%	45.86%	68.79%	Beta	(12)
Nivolumab plus ipilimumab	53.54%	42.83%	64.25%	Beta	(12)
**Others**					
Discount rate	5.00%	0.00%	8.00%	Fixed	(17)
Patient weight (kg)	65.00	52.00	78.00	Gamma	(25)
Body surface area (m^2^)	1.72	1.38	2.06	Gamma	(25)

ESCC, esophageal squamous-cell carcinoma; PFS, progression-free survival; OS, overall survival.

### Scenario analysis

Our analyses covered two scenarios. In the first scenario, we assumed that nivolumab and ipilimumab were reduced to 80%, 60%, 40% or 20% of the current price to explore the cost-effectiveness of nivolumab combination therapy, respectively. In addition, we evaluated the impact of a longer or shorter time horizon of simulation on ICERs.

### Sensitivity analyses

In order to evaluate the robustness of the model and identify the variables that have considerable impacts on the analysis results, we performed one-way and probabilistic sensitivity analyses (PSA) for input parameters. In the one-way sensitivity analysis, input parameters were adjusted one-by-one to their respective minimum and maximum values, with a range of the 95% confidence intervals reported in the referenced literature or a ± 20% change from the base-case value, in order to ascertain the variables that significantly influenced the economic outcomes. The range of discount rate was 0%-8%. Tornado diagram was used to present the results. A Monte Carlo simulation of 10,000 iterations was conducted for PSA by simultaneously sampling all input parameters from the pre-specified distributions. All the costs were sampled from Gamma distribution. The utility values and probabilities were sampled from Beta distribution. Cost-effectiveness acceptability curves (CEAC) were plotted based on the outcomes from 10,000 iterations to illustrate the probability of cost-effectiveness of nivolumab plus chemotherapy and nivolumab plus ipilimumab against chemotherapy alone at various WTP thresholds.

## Results

### Base-case results

The base-case results are presented in **Table 3**. Over the lifetime horizon of 10 years, compared with chemotherapy, nivolumab plus chemotherapy or ipilimumab as first-line therapy for overall advanced ESCC patients provided an incremental cost of $78,349.01 and $63,058.82 with additional 0.19 QALYs and 0.15 QALYs, respectively, resulting in an ICER of $415,163.81/QALY and $430,704.11/QALY. Compared with chemotherapy, nivolumab plus chemotherapy or ipilimumab as first-line therapy for PD-L1-positive advanced ESCC patients generated an incremental cost of $ 88,366.61 and $ 89,257.72 with additional 0.41 QALYs and 0.48 QALYs, respectively, resulting in an ICER of $216,628.00/QALY and $185,483.94/QALY. In the pairwise comparison between the two nivolumab combination therapies, nivolumab plus ipilimumab increased the cost by $891.12 with the augments of 0.07 QALYs against nivolumab plus chemotherapy for PD-L1-positive advanced ESCC patients, and the ICER ($12,157.66/QALY) was lower than the WTP threshold.

**Table 3:** Base case results.

	Overall advanced ESCC patients			Advanced ESCC patients with PD-L1-positive status		
Parameters	Chemotherapy	Nivolumab plus chemotherapy	Nivolumab plus ipilimumab	Chemotherapy	Nivolumab plus chemotherapy	Nivolumab plus ipilimumab
**Cost ($)**						
Drug	6,952.79	88,285.53	72,568.91	5,781.46	96,689.13	97,103.71
Follow-up and tests	1,009.81	1,439.20	1,069.46	839.69	1,602.10	1,594.97
Adverse events	29.13	35.22	6.69^#^	24.22	39.33	14.10^#^
PFS state	7,991.72	89,759.94	73,645.06	6,645.37	98,330.56	98,712.79
PD state	5,047.23	1,628.02	2,452.71	5,605.15	2,286.57	2,795.46
Terminal care	1,460.30	1,460.30	1,460.30	1,460.30	1,460.30	1,460.30
Total Cost	14,499.25	92,848.26	77,558.07	13,710.82	102,077.43	102,968.54
**LYs**						
PFS state	0.62	0.91	0.68	0.51	1.02	1.04
PD state	0.45	0.45	0.69	0.84	1.13	1.53
Total LYs	1.08	1.36	1.38	1.35	2.15	2.57
**QALYs**						
PFS state	0.45	0.64	0.48	0.37	0.72	0.71
PD state	0.24	0.24	0.36	0.27	0.34	0.41
Total QALYs	0.70	0.88	0.84	0.65	1.05	1.13
**ICER ($/LYs)**		272,390.06	208,386.78		110,465.61	73,402.85
			-1,021,434.44^*^			2,141.84^*^
**ICER ($/QALY)**		415,163.81	430,704.11		216,628.00	185,483.94
			361,388.00^*^			12,157.66^*^

^#^Management costs associated with adverse events caused by chemotherapy after two years of treatment with nivolumab plus ipilimumab; *nivolumab plus chemotherapy versus nivolumab plus ipilimumab; ESCC, esophageal squamous-cell carcinoma; PFS, progression-free survival; OS, overall survival; LYs, lifeyears; QALYs, Quality-adjusted life years; ICER, Incremental cost-effectiveness ratio.

### Scenario analysis results

The results of the scenario analysis are shown in **Supplementary Tables 4, 5**. As the price of nivolumab and ipilimumab decreased or the time horizon of simulation increased, the ICER of nivolumab combination therapy over chemotherapy gradually decreased. With 80% price reduction of nivolumab and ipilimumab, the ICER ($29,649.50/QALY) of nivolumab plus ipilimumab versus chemotherapy was below the WTP threshold in the treatment of PD-L1-positive advanced ESCC patients.

### One-way sensitivity analysis

**Figure 2:**
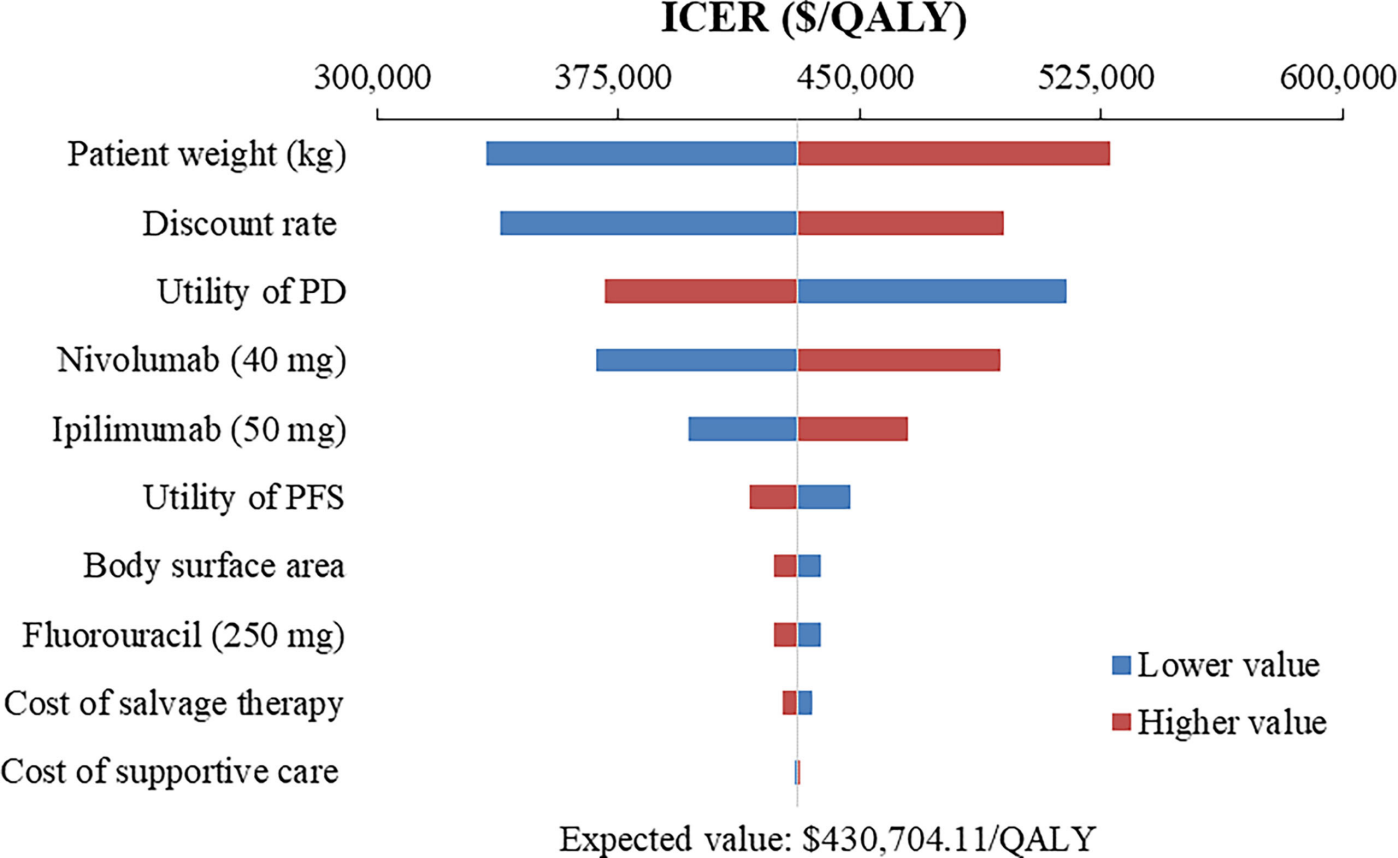
Tornado diagram of one-way sensitivity analysis of Nivolumab plus ipilimumab versus Chemotherapy in the treatment of overall advanced ESCC patients. ICER, incremental cost-effectiveness ratio; QALY, quality-adjusted life year; PFS, progression-free survival; PD, progressive disease.

**Figure 3:**
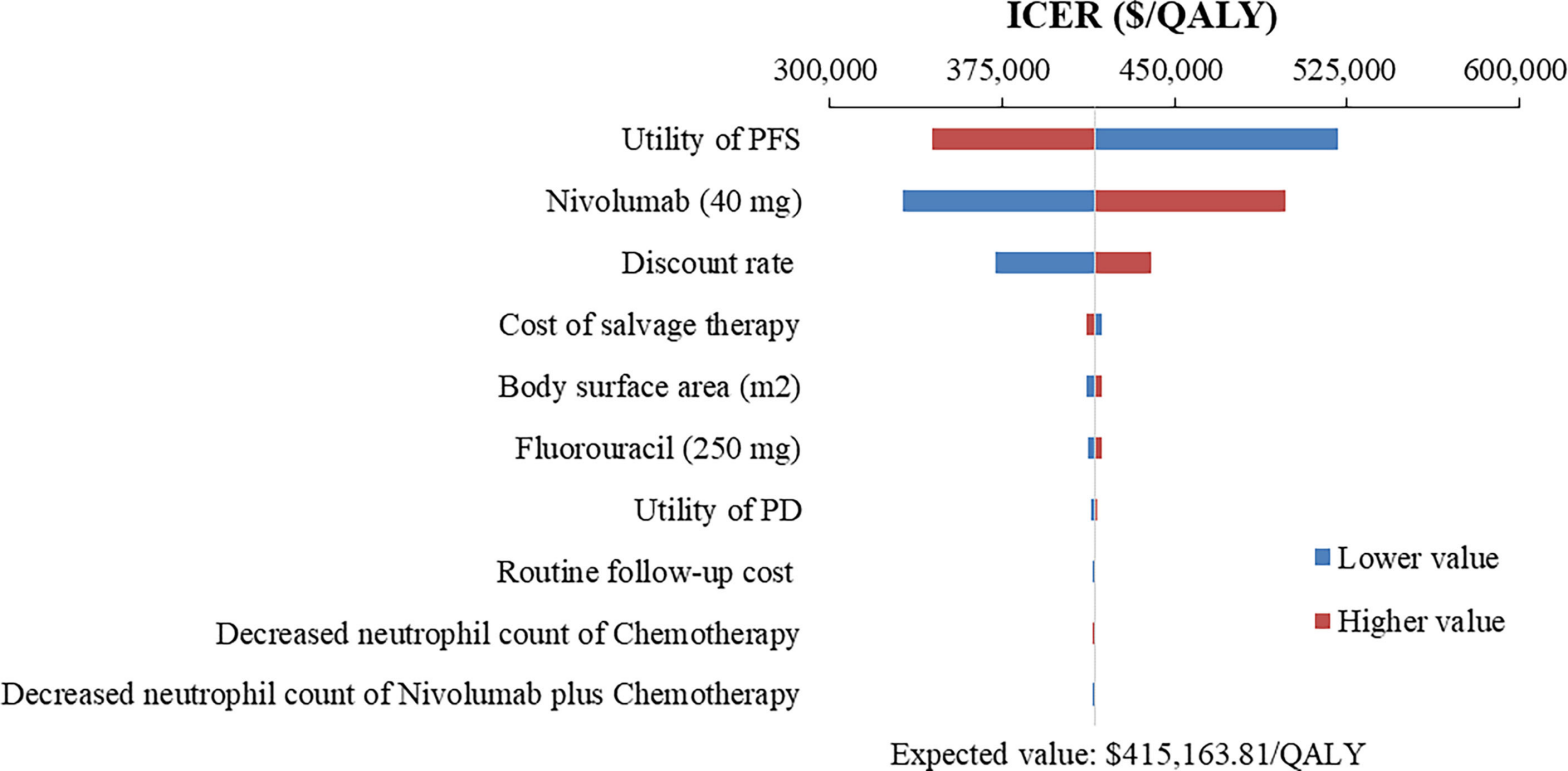
Tornado diagram of one-way sensitivity analysis of Nivolumab plus chemotherapy versus Chemotherapy in the treatment of overall advanced ESCC patients. ICER, incremental cost-effectiveness ratio; QALY, quality-adjusted life year; PFS, progression-free survival; PD, progressive disease.

**Figure 4:**
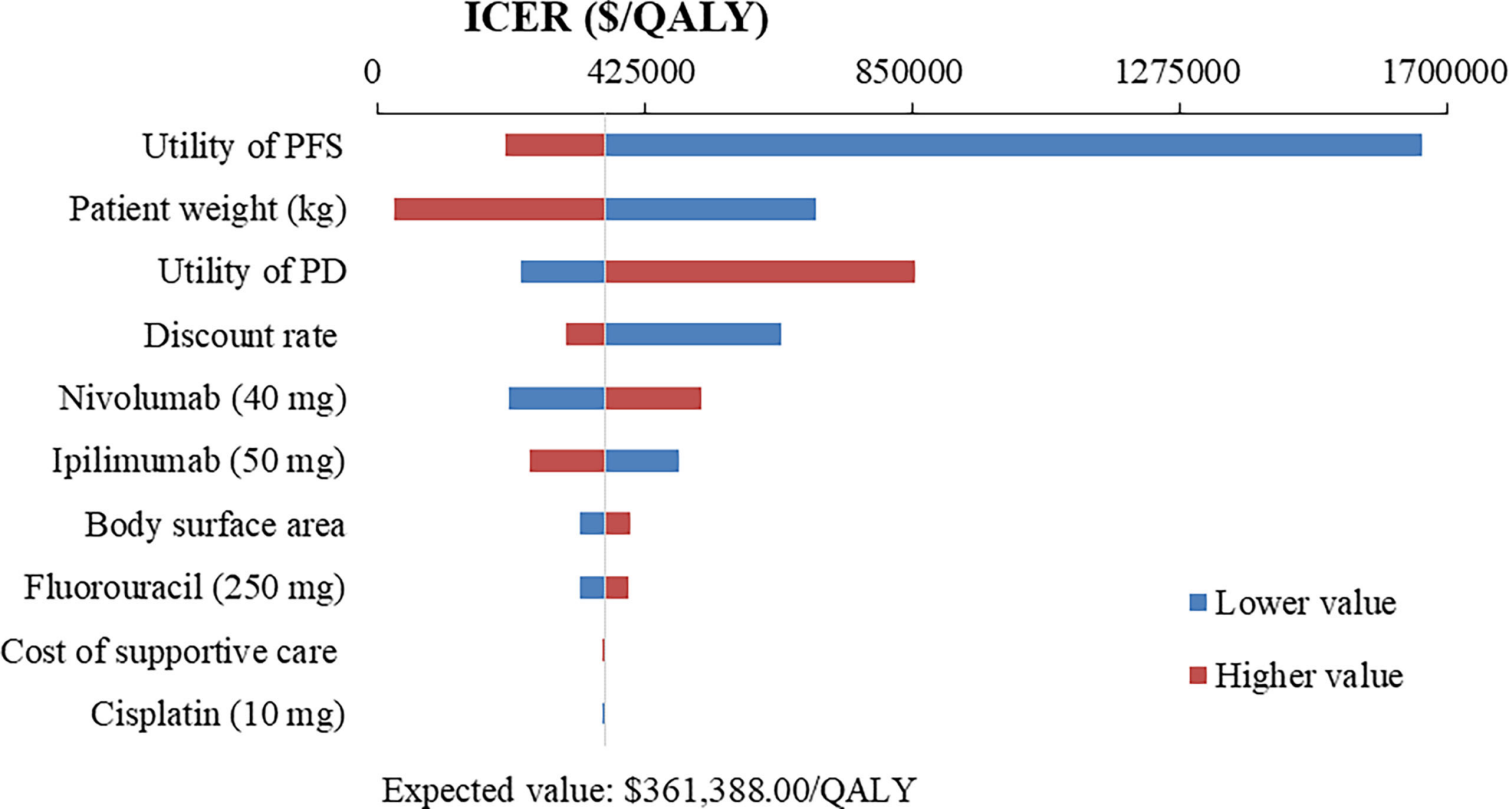
Tornado diagram of one-way sensitivity analysis of Nivolumab plus chemotherapy versus Nivolumab plus ipilimumab in the treatment of overall advanced ESCC patients. ICER, incremental cost-effectiveness ratio; QALY, quality-adjusted life year; PFS, progression-free survival; PD, progressive disease.

The top 10 parameters that most influenced the base-case analysis of overall advanced ESCC patients are presented in Tornado diagrams (**Figures 2**–**4**). Patients’ weight, utility values, and the prices of nivolumab and ipilimumab greatly influenced the model results. Similar results were obtained in PD-L1-positive advanced ESCC patients (**Supplementary Figures 13–15**).

### Probabilistic sensitivity analysis

**Figure 5:**
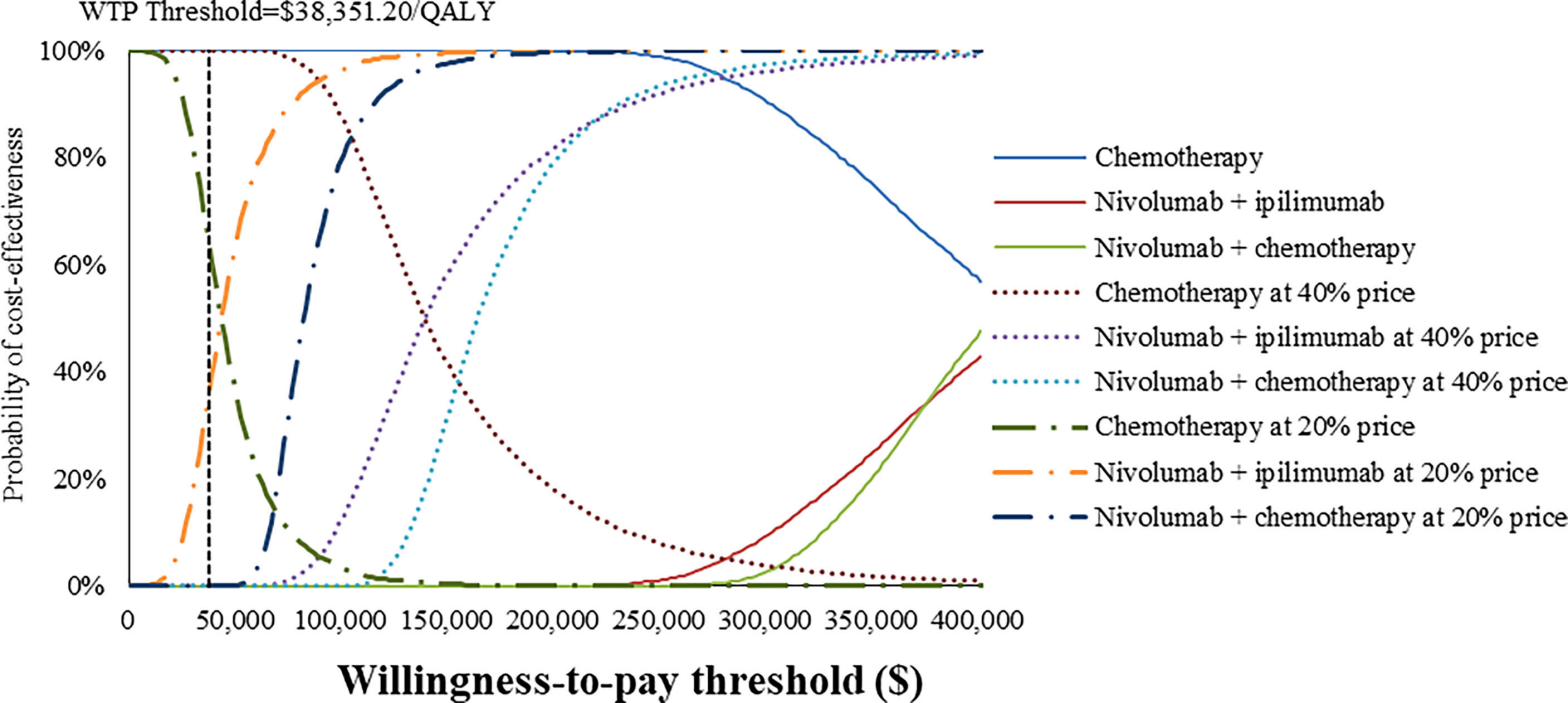
Cost-effectiveness acceptability curves of nivolumab plus ipilimumab and nivolumab plus chemotherapy versus chemotherapy in the treatment of overall advanced ESCC patients from the Chinese healthcare perspective.

**Figure 6:**
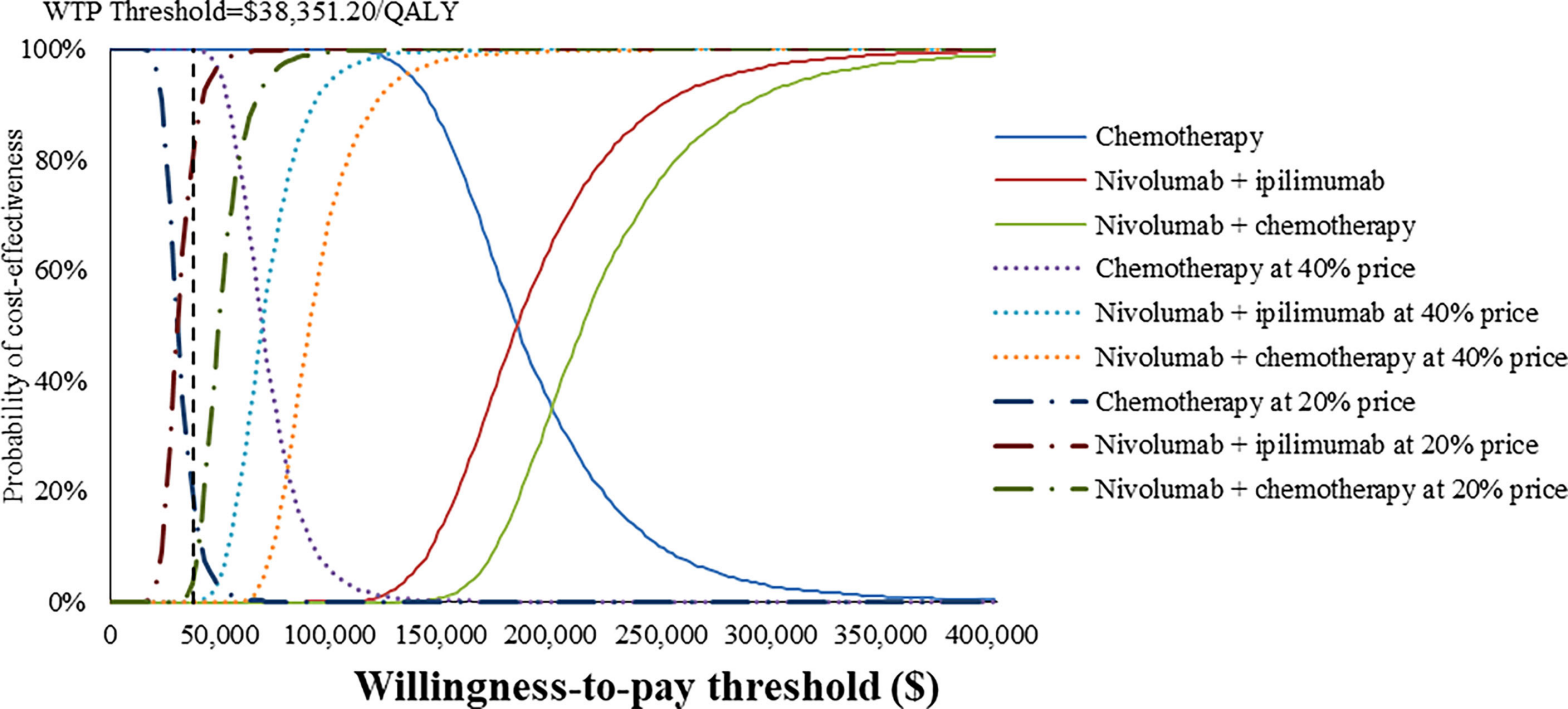
Cost-effectiveness acceptability curves of nivolumab plus ipilimumab and nivolumab plus chemotherapy versus chemotherapy in the treatment of PD-L1-positive advanced ESCC patients from the Chinese healthcare perspective.

At the base-case WTP threshold and current price, the CEAC demonstrated that the probability of nivolumab combination therapy strategies being cost-effective was 0% in overall and PD-L1-positive ESCC patients (**Figures 5**, **6**). As the price of nivolumab and ipilimumab decreased, the results of the PSA have changed. When the price of nivolumab and ipilimumab reduced 80%, the probability of being cost-effective increased to 0% and 7.85% for nivolumab plus chemotherapy and 40.44% and 86.38% for nivolumab plus ipilimumab in overall and PD-L1-positive advanced ESCC patients, respectively. In the pairwise comparison between the two nivolumab combination therapies, the probability of nivolumab plus chemotherapy being cost-effectiveness was 4.72% and 41.26% in overall and PD-L1-positive advanced ESCC patients at the WTP threshold of $38,351.20 per QALY, respectively, compared with nivolumab plus ipilimumab (**Supplementary Figures 16, 17**).

## Discussion

To our knowledge, this study was the first modeling analysis to examine the cost-effectiveness of nivolumab combination therapy in the treatment of advanced ESCC patients by incorporating the latest evidence from a Chinese healthcare system perspective. The results revealed that nivolumab combination therapy could provide higher health outcomes with higher cost expenditures, the ICER well above the WTP threshold based on the latest GDP. Sensitivity analyses confirmed that the model results were robust. Considering the implementation of the national price negotiation policy in China (36, 37), we assumed that nivolumab and ipilimumab were reduced to 40% or 20% of the current price, respectively, to explore the optimal treatment options. The results of PSA indicated that when the price of nivolumab and ipilimumab at 20% price, the cost-effective probability of nivolumab plus ipilimumab improved from 0% to 40.44% and 86.38% in overall and PD-L1-positive advanced ESCC patients, respectively, otherwise chemotherapy was dominant at a WTP threshold of $38,351.20/QALY.

Regardless of the overall or PD-L1-positive advanced ESCC patients, nivolumab combined with chemotherapy or ipilimumab yielded near-equal health outcomes over a 10-year lifetime horizon estimation. In the PFS state, the QALYs produced by nivolumab plus ipilimumab were much lower than that of nivolumab plus chemotherapy for overall advanced ESCC patients, while there was almost identity between the two treatment regiments for PD-L1 positive ESCC patients. In the PD state, with the increase of time horizon, nivolumab plus ipilimumab could accumulate more QALYs than nivolumab plus chemotherapy, which benefited from the improvement of overall survival time. As such, the cost-effectiveness advantage of nivolumab plus ipilimumab compared with nivolumab plus chemotherapy progressively emerged as the simulation time increased. It was worth mentioning that these results should be interpreted with caution, due to the lack of sufficient data on the cost and disutility values of treatment-related AEs in the nivolumab plus ipilimumab group.

Due to the dramatically increasing cost and the uncertainty of survival benefits, innovative drugs combined with existing treatment schemes often have lower cost-effective probabilities than standard treatment regimens (14). Although the survival benefits of nivolumab combination therapy were superior to chemotherapy in the treatment of advanced ESCC, the higher expenditures and limited improvement in health outcomes were such that substantial price reductions still could not salvage its cost-effectiveness. Previous economic evidence suggested that nivolumab was not a cost-effective treatment option compared with chemotherapy in the second-line treatment of advanced ESCC patients from the perspective of Chinese society (15, 38). Our findings were consistent with those of previous economic evaluations, and the total cost and QALYs were different, which might be caused by various treatment schedules, modeling techniques, and cost measurements used in the two studies.

Among patients with advanced ESCC, the addition of camrelizumab (an anti-PD-1 antibody) to chemotherapy also significantly improved PFS (6.9 vs. 5.6 months; HR for progression or death, 0.56; 95% CI, 0.46-0.68; P<0.001) and OS (15.3 vs. 12.0 months; HR for death, 0.70; 95% CI, 0.56-0.88; P=0.001) in comparison with single-agent chemotherapy (39). Similarly, the latest cost-effectiveness analysis has shown that camrelizumab plus chemotherapy was unlikely to be cost-effective versus chemotherapy in patients with advanced or metastatic ESCC over a 5-year lifetime horizon estimation in China (27). However, after a price reduction of 85.2% through China’s drug price negotiation mechanism, camrelizumab was a cost-effective treatment regimen against chemotherapy for advanced or metastatic ESCC patients (35). Consequently, in the absence of further breakthroughs in efficacy at this time, a substantial price reduction is the key to ensuring cost-effectiveness and affordability of treatment options, especially in countries with a huge cancer burden and limited medical resources (40). Our sensitivity analyses also indicated that drug price was an important variable affecting ICER, and price reduction could improve the cost-effective probability of nivolumab combination therapy. In addition, equitable and niche-targeting PAP can yet be regarded as a shortcut to improve affordability.

In addition to the price of nivolumab and ipilimumab, sensitivity analyses demonstrated that patients’ weight and utility values for PFS and PD state were the most influential parameter within the model. We used the default body weight to estimate the dosage of the therapeutic agents in the base-case analysis, which limited the transferability and representativeness of specific population, such as the over-weight (41, 42). Therefore, weight-specific economic evaluations warranted further studies to best inform cancer precision medicine and reimbursement policy (43). Furthermore, the quality of life research of esophageal neoplasms has been available in China (44, 45), but these still cannot meet the urgent needs of health technology assessment, especially the lack of utility and disutility values associated with various health states and treatment regimens. Hence, developing health utility values based on realistic modeling needs remains a priority.

As model assumptions and limited data, several potential limitations should be considered in the current economic evaluation. First, we reconstructed IPD rather than actual data from the CheckMate 648 trial because the original data were unavailable from the published literature. Although this approach was not perfect, it approximately reflected the actual survival data observed in the clinical trials so as to guarantee the credibility of this simulation. Second, since the quality of life was not reported in the CheckMate 648 trial, we obtained utility values from the published literature. That might lead to some deviations between the simulation results and actual health outcomes. Therefore, we used a wide range (± 20%) of utility values to examine the effect of changes on outcomes in the sensitivity analysis, which did not substantially impact the base-case results. Third, we only considered disutility values and costs related to grade 3-4 AEs of chemotherapy and nivolumab plus chemotherapy group, as these were difficult to define and obtain in the nivolumab plus ipilimumab group. Fourth, some important cost variables were derived from published economic evaluations rather than the real-world medical data, although one-way sensitivity analysis proved that these costs exerted minimal influence on the model results, except for the costs of nivolumab and ipilimumab. Fifth, we assumed that the best supportive care was administrated after the progression of nivolumab combination therapy, which might differ from the actual treatment options.

## Conclusion

In summary, nivolumab combination therapy was unlikely to be a cost-effective treatment regimen compared with chemotherapy in the first-line treatment of patients with advanced ESCC in China. When the price of nivolumab and ipilimumab decreased 80%, nivolumab plus ipilimumab was the optimal treatment option among PD-L1-positive advanced ESCC patients in China.

## Data Availability Statement

The original contributions presented in the study are included in the article/**Supplementary Material**. Further inquiries can be directed to the corresponding author.

## Author Contributions

SXL, SPL, and LD were responsible for study design, model building and statistical analysis. SXL prepared the manuscript. KW, ZS, RW, XZ, and ZHS searched literatures and collected data. All authors critically reviewed the model structure, verified results and revised the manuscript.

## Conflict of Interest

The authors declare that the research was conducted in the absence of any commercial or financial relationships that could be construed as a potential conflict of interest.

## Publisher’s Note

All claims expressed in this article are solely those of the authors and do not necessarily represent those of their affiliated organizations, or those of the publisher, the editors and the reviewers. Any product that may be evaluated in this article, or claim that may be made by its manufacturer, is not guaranteed or endorsed by the publisher.
