# Supplementary material for: Cost-effectiveness analysis of nivolumab combination therapy in the first-line treatment for advanced esophageal squamous-cell carcinoma
Source: Front Oncol. 2022 Jul 22;12:899966. doi: 10.3389/fonc.2022.899966 (PMC9353037; doi:10.3389/fonc.2022.899966)
Supplement: Supplementary file 1 [file DataSheet_1.zip › Additional files/Supplementary Figures.pdf]

Supplementary Figures

Supplementary Figure 1 | The reconstructed Kaplan-Meier PFS curves of chemotherapy regimen in overall population

Supplementary Figure 2 | The reconstructed Kaplan-Meier OS curves of chemotherapy regimen in overall population

Supplementary Figure 3 | The reconstructed Kaplan-Meier PFS curves of nivolumab plus chemotherapy regimen in overall population

Supplementary Figure 4 | The reconstructed Kaplan-Meier OS curves of nivolumab plus chemotherapy regimen in overall population

Supplementary Figure 5 | The reconstructed Kaplan-Meier PFS curves of Nivolumab plus ipilimumab regimen in overall population

Supplementary Figure 6 | The reconstructed Kaplan-Meier OS curves of Nivolumab plus ipilimumab regimen in overall population

Supplementary Figure 7 | The reconstructed Kaplan-Meier PFS curves of chemotherapy regimen in tumor-cell pd-l1 expression of ⩾1% patients

Supplementary Figure 8 | The reconstructed Kaplan-Meier OS curves of chemotherapy regimen in tumor-cell pd-l1 expression of ⩾1% patients

Supplementary Figure 9 | The reconstructed Kaplan-Meier PFS curves of nivolumab plus chemotherapy regimen in tumor-cell pd-l1 expression of ⩾1% patients

Supplementary Figure 10 | The reconstructed Kaplan-Meier OS curves of nivolumab plus chemotherapy regimen in tumor-cell pd-l1 expression of ⩾1% patients

Supplementary Figure 11 | The reconstructed Kaplan-Meier PFS curves of nivolumab plus ipilimumab regimen in tumor-cell pd-l1 expression of ⩾1% patients

Supplementary Figure 12 | The reconstructed Kaplan-Meier OS curves of nivolumab plus ipilimumab regimen in tumor-cell pd-l1 expression of ⩾1% patients

Supplementary Figure 13 | Tornado diagram of one-way sensitivity analysis of Nivolumab plus ipilimumab versus Chemotherapy in the treatment of PD-L1-positive advanced ESCC patients. ICER, incremental cost-effectiveness ratio; QALY, quality-adjusted life year; PFS, progression-free survival; PD, progressive disease.

Supplementary Figure 14 | Tornado diagram of one-way sensitivity analysis of Nivolumab plus chemotherapy versus Chemotherapy in the treatment of PD-L1-positive advanced ESCC patients. ICER, incremental cost-effectiveness ratio; QALY, quality-adjusted life year; PFS, progression-free survival; PD, progressive disease.

Supplementary Figure 15 | Tornado diagram of one-way sensitivity analysis of Nivolumab plus ipilimumab versus Nivolumab plus chemotherapy in the treatment of PD-L1-positive advanced ESCC patients. ICER, incremental cost-effectiveness ratio; QALY, quality-adjusted life year; PFS, progression-free survival; PD, progressive disease.

Supplementary Figure 16 | Cost-effectiveness acceptability curves of nivolumab plus ipilimumab versus nivolumab plus chemotherapy in the treatment of overall advanced ESCC patients from the Chinese healthcare perspective.

Supplementary Figure 17 | Cost-effectiveness acceptability curves of nivolumab plus ipilimumab versus nivolumab plus chemotherapy in the treatment of PD-L1-positive advanced ESCC patients from the Chinese healthcare perspective.

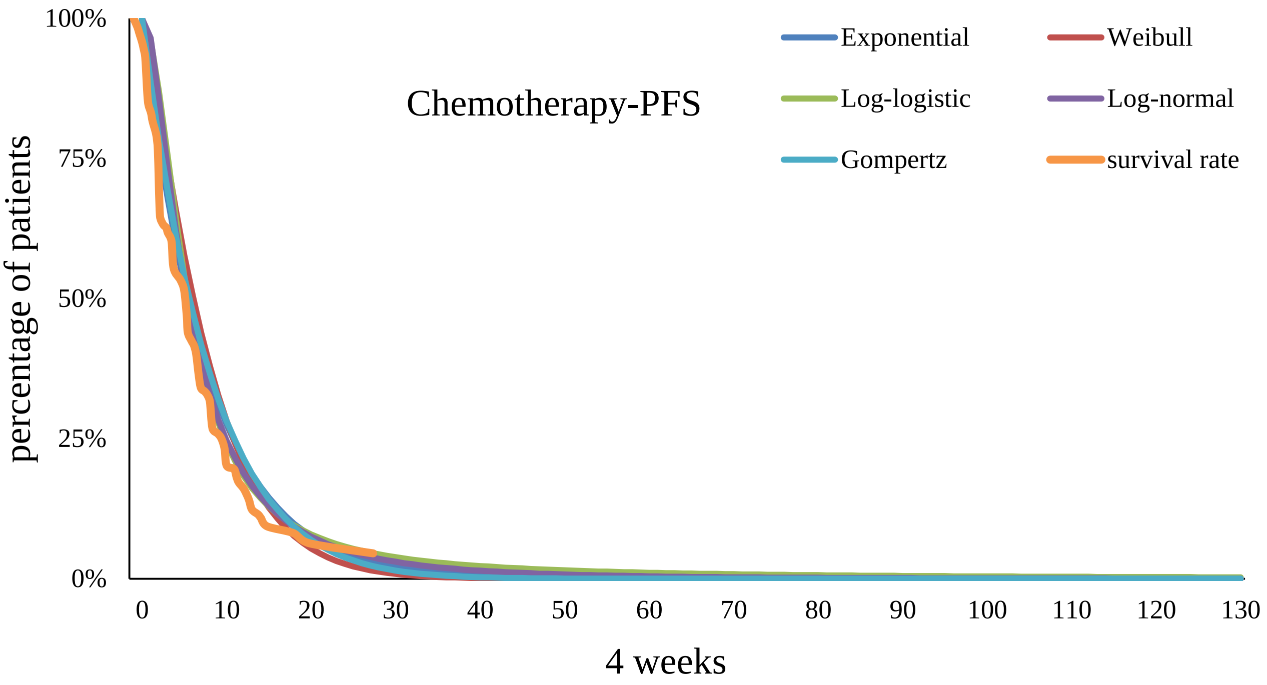

Supplementary Figure 1 | The reconstructed Kaplan-Meier PFS curves of chemotherapy regimen in overall population

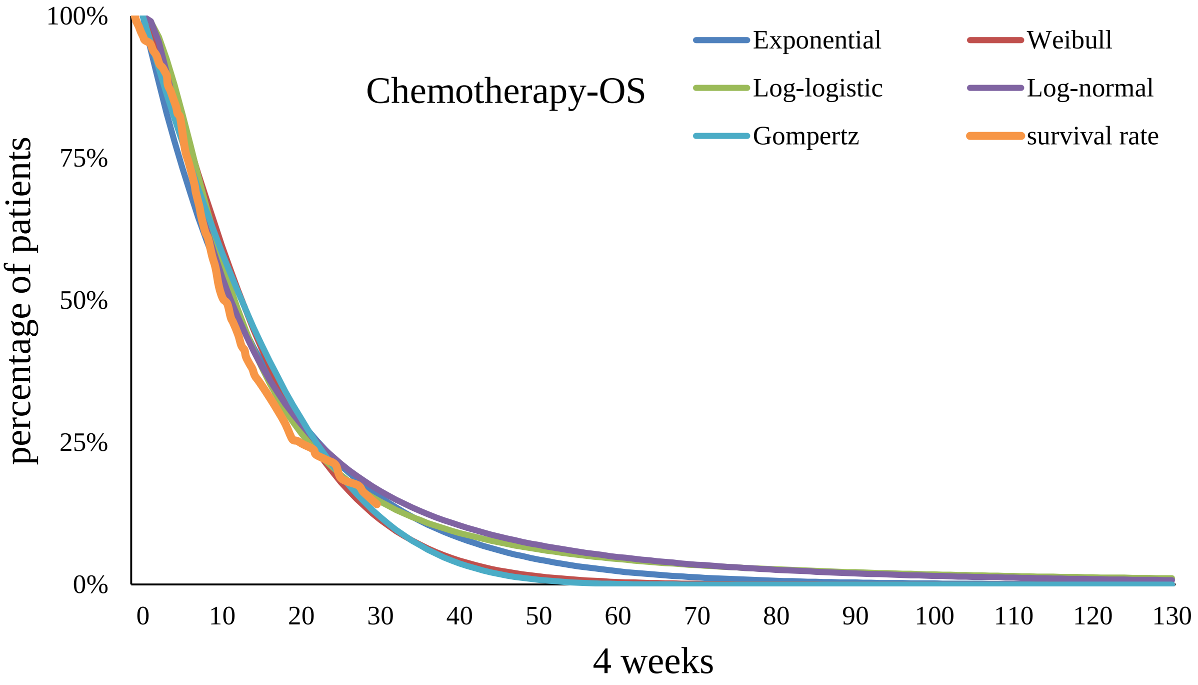

Supplementary Figure 2 | The reconstructed Kaplan-Meier OS curves of chemotherapy regimen in overall population

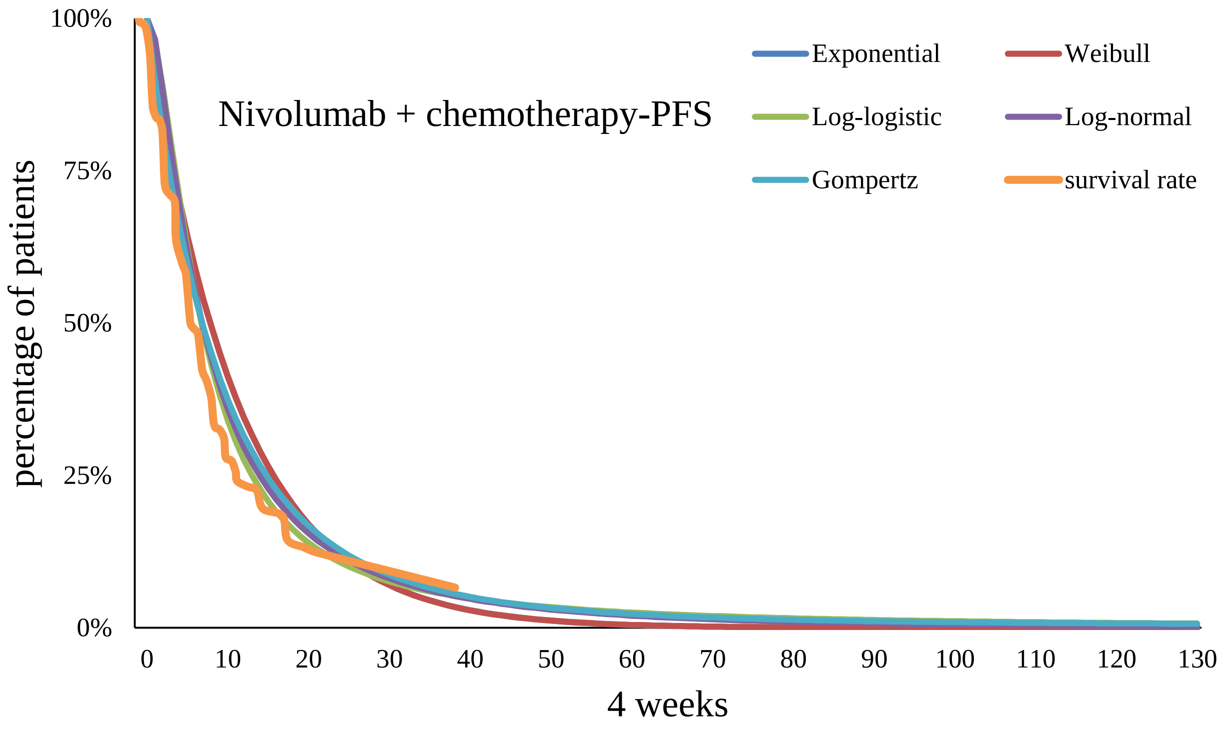

Supplementary Figure 3 | The reconstructed Kaplan-Meier PFS curves of nivolumab plus chemotherapy regimen in overall population

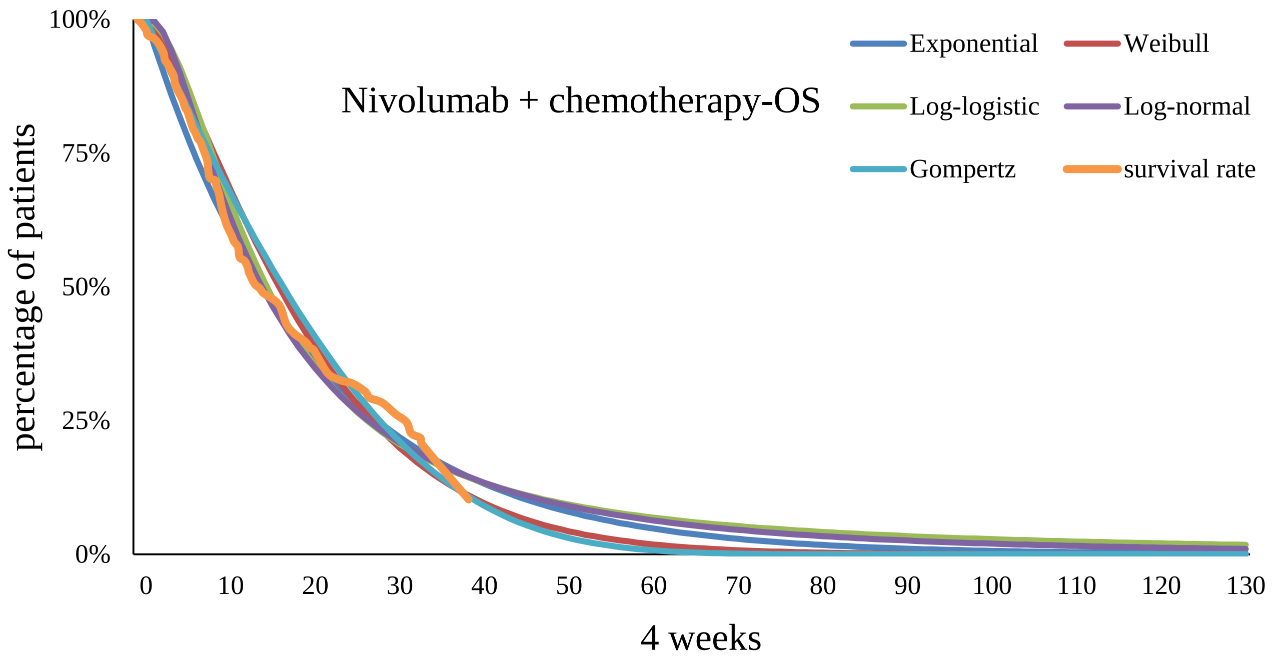

Supplementary Figure 4 | The reconstructed Kaplan-Meier OS curves of nivolumab plus chemotherapy regimen in overall population

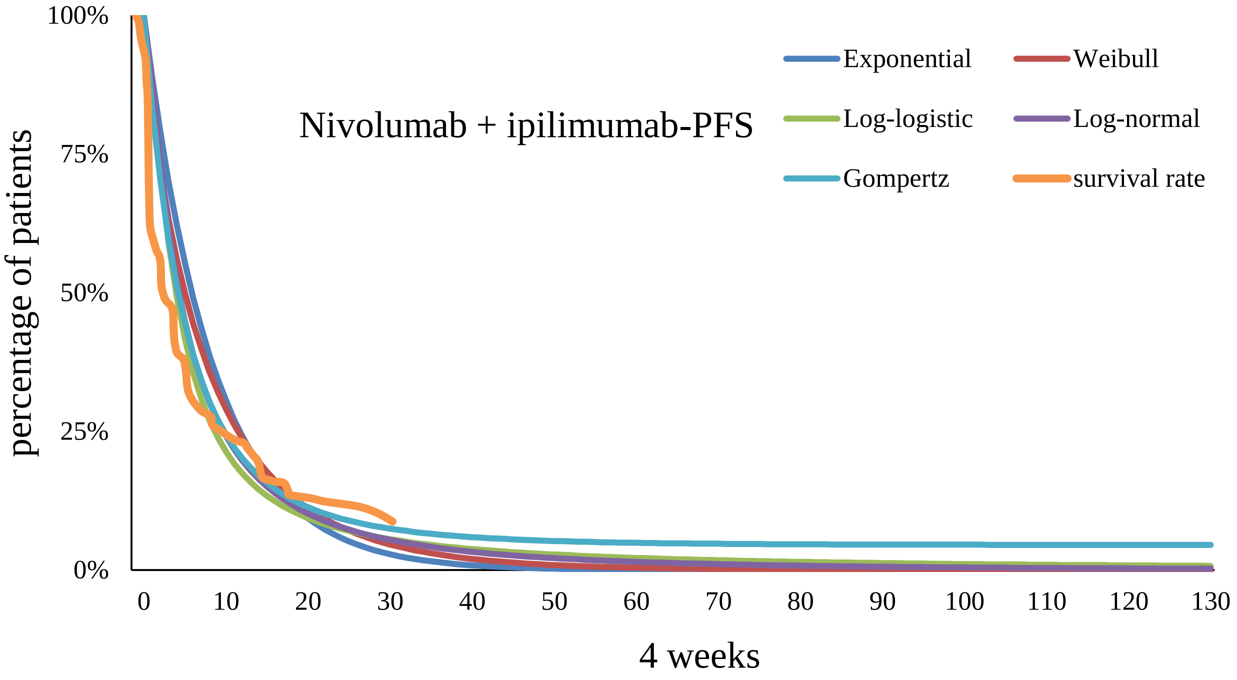

Supplementary Figure 5 | The reconstructed Kaplan-Meier PFS curves of Nivolumab plus ipilimumab regimen in overall population

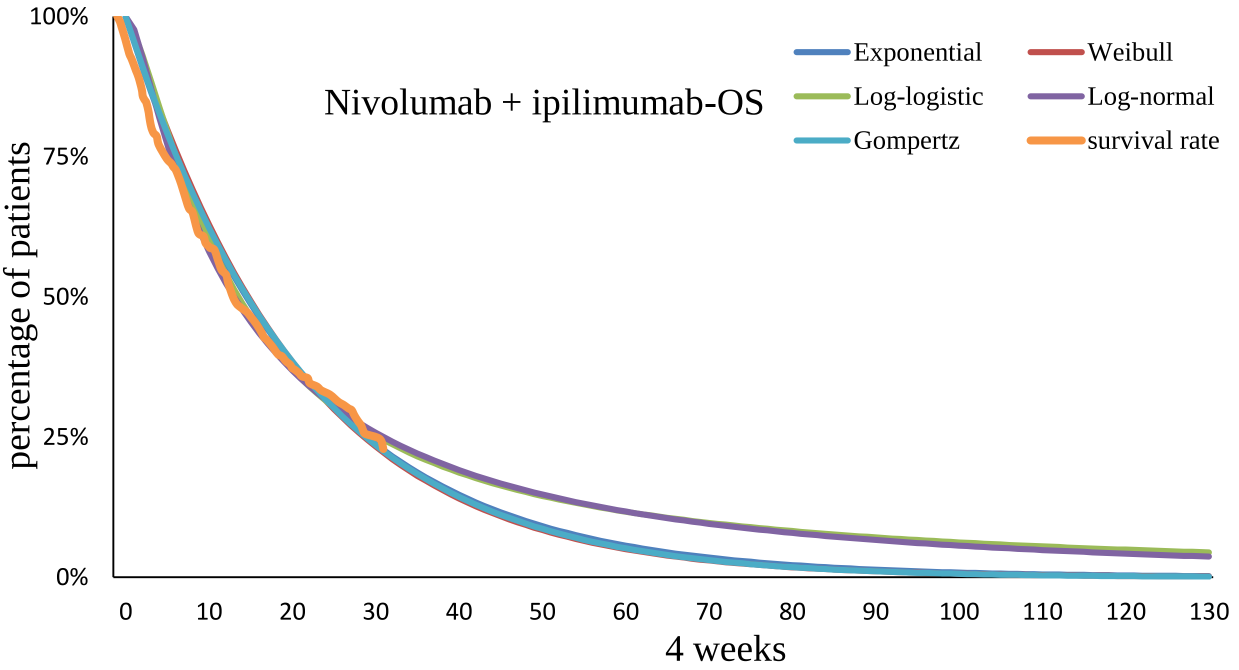

Supplementary Figure 6 | The reconstructed Kaplan-Meier OS curves of Nivolumab plus ipilimumab regimen in overall population

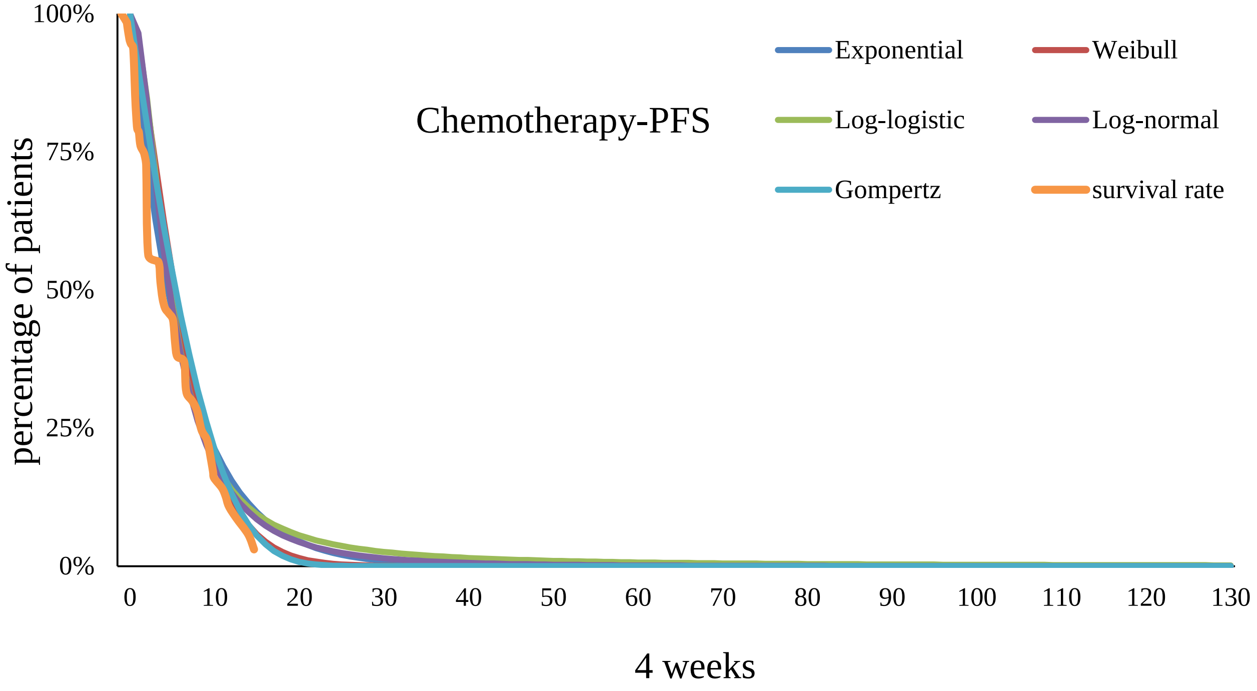

Supplementary Figure 7 | The reconstructed Kaplan-Meier PFS curves of chemotherapy regimen in tumor-cell pd-l1 expression of ⩾1% patients

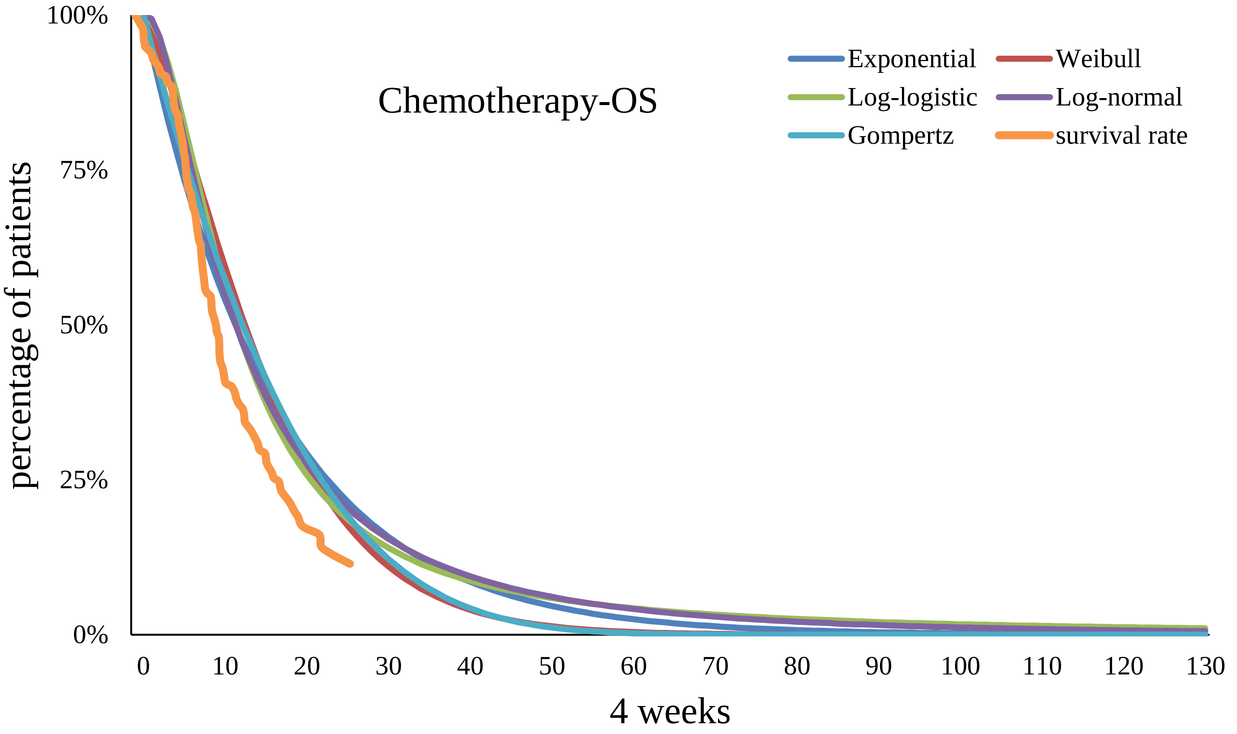

Supplementary Figure 8 | The reconstructed Kaplan-Meier OS curves of chemotherapy regimen in tumor-cell pd-l1 expression of ⩾1% patients

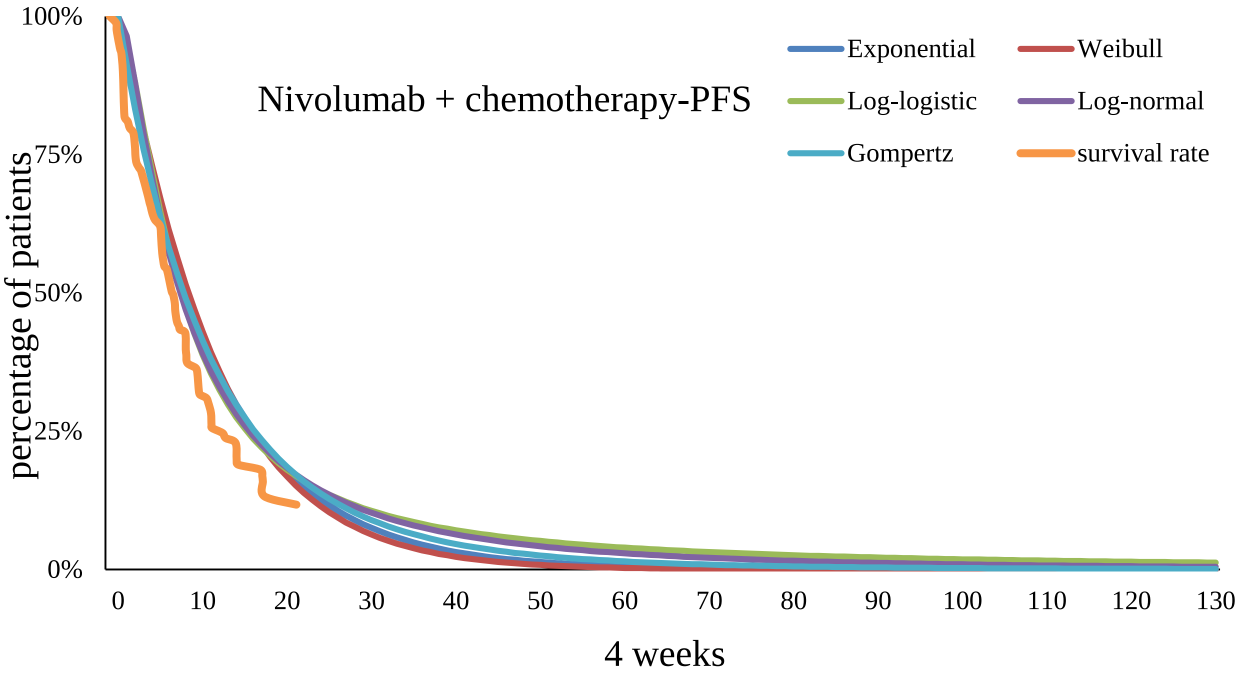

Supplementary Figure 9 | The reconstructed Kaplan-Meier PFS curves of nivolumab plus chemotherapy regimen in tumor-cell pd-l1 expression of ⩾1% patients

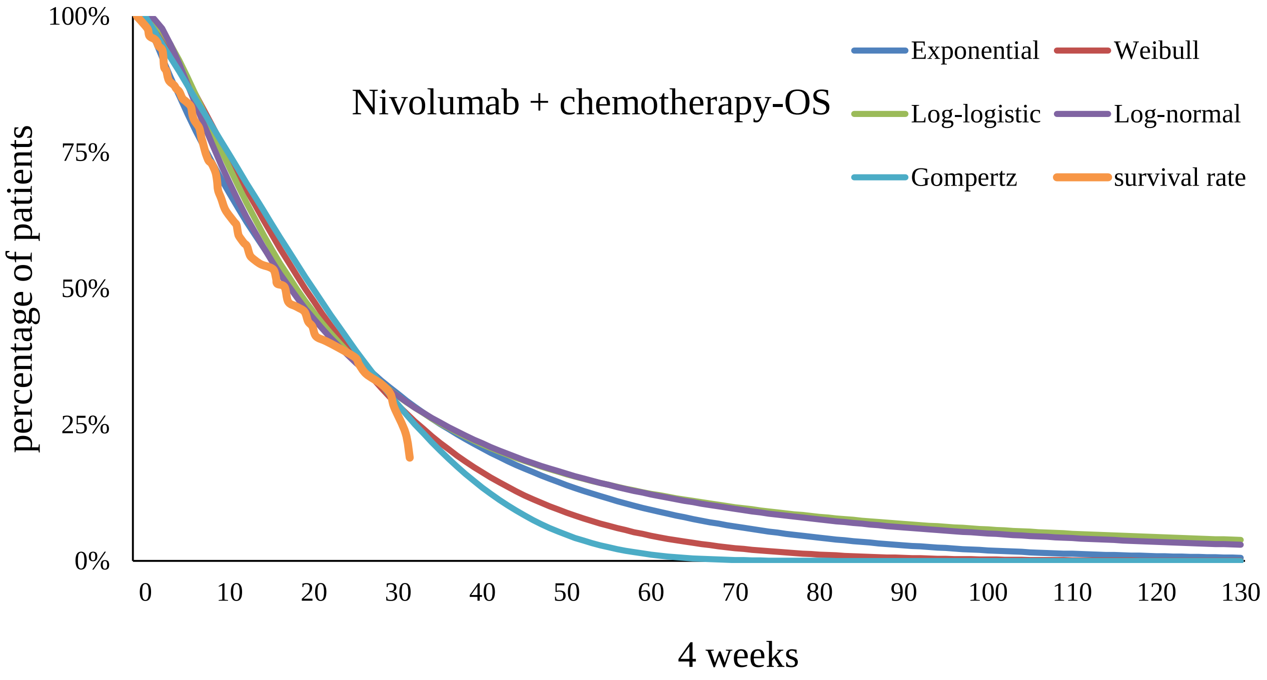

Supplementary Figure 10 | The reconstructed Kaplan-Meier OS curves of nivolumab plus chemotherapy regimen in tumor-cell pd-l1 expression of ⩾1% patients

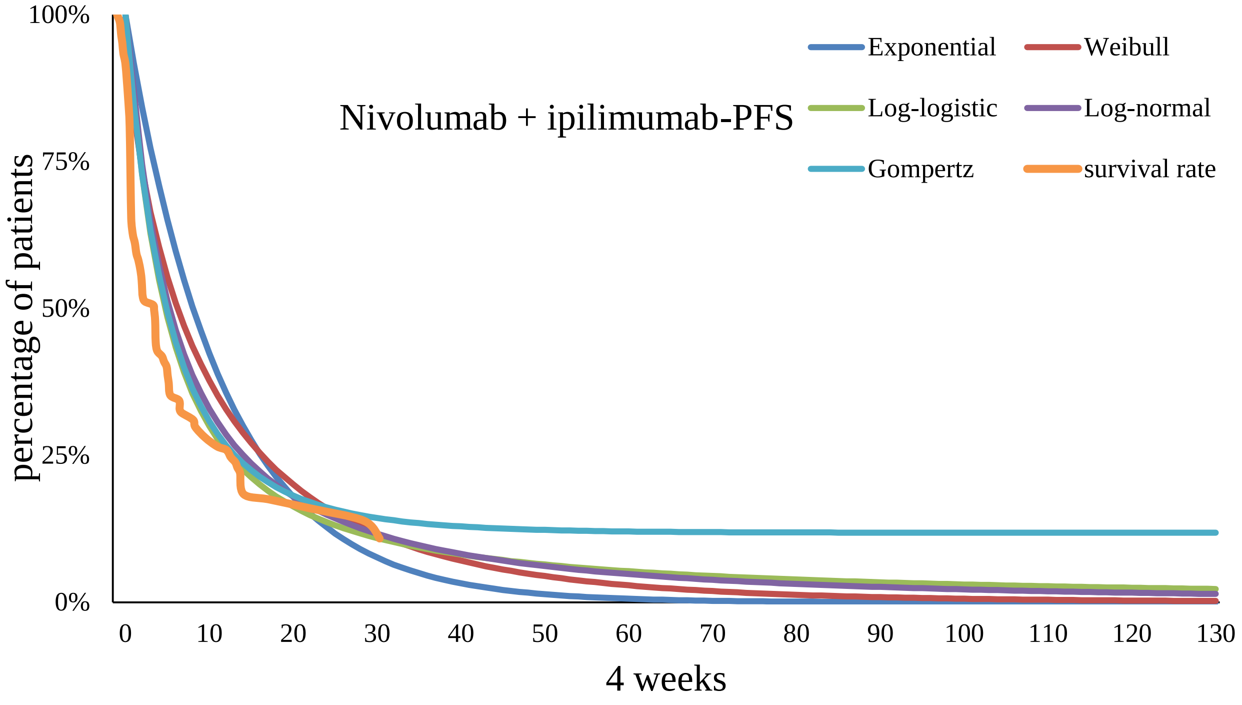

Supplementary Figure 11 | The reconstructed Kaplan-Meier PFS curves of nivolumab plus ipilimumab regimen in tumor-cell pd-l1 expression of ⩾1% patients

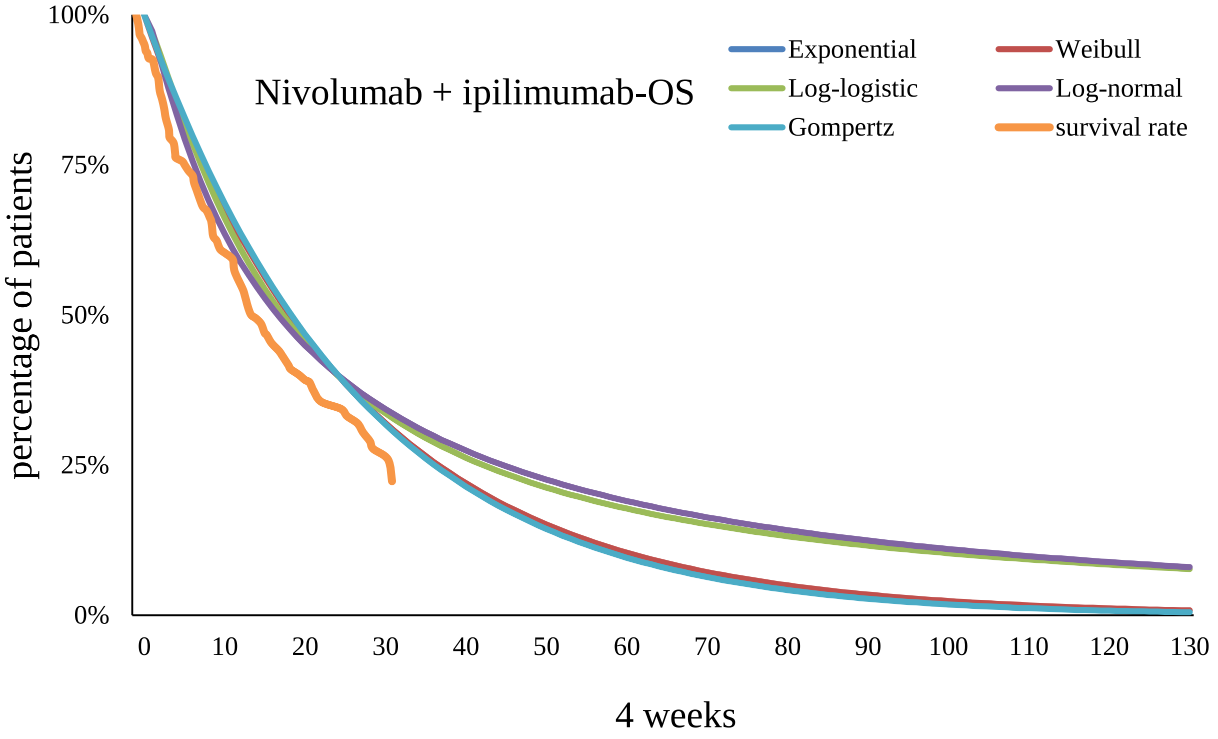

Supplementary Figure 12 | The reconstructed Kaplan-Meier OS curves of nivolumab plus ipilimumab regimen in tumor-cell pd-l1 expression of ⩾1% patients

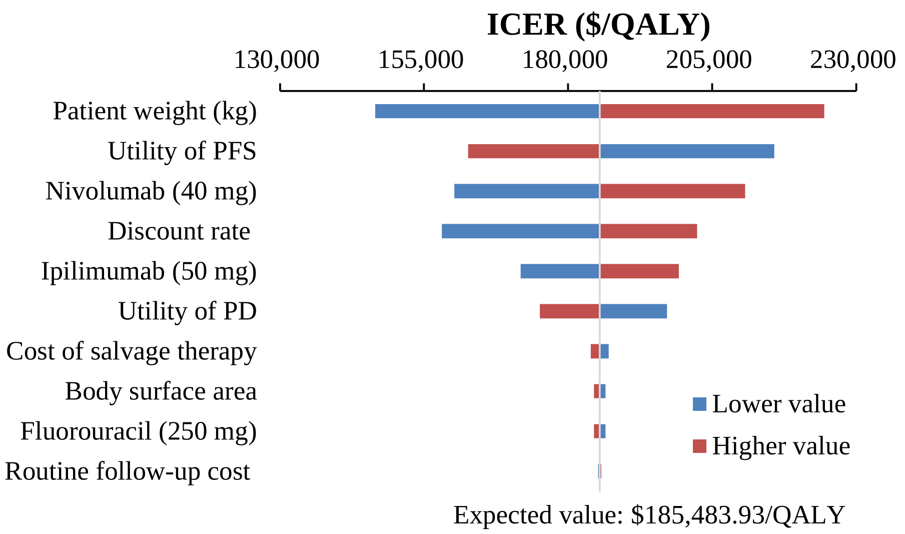

Supplementary Figure 13 | Tornado diagram of one-way sensitivity analysis of Nivolumab plus ipilimumab versus Chemotherapy in the treatment of PD-L1-positive advanced ESCC patients. ICER, incremental cost-effectiveness ratio; QALY, quality-adjusted life year; PFS, progression-free survival; PD, progressive disease.

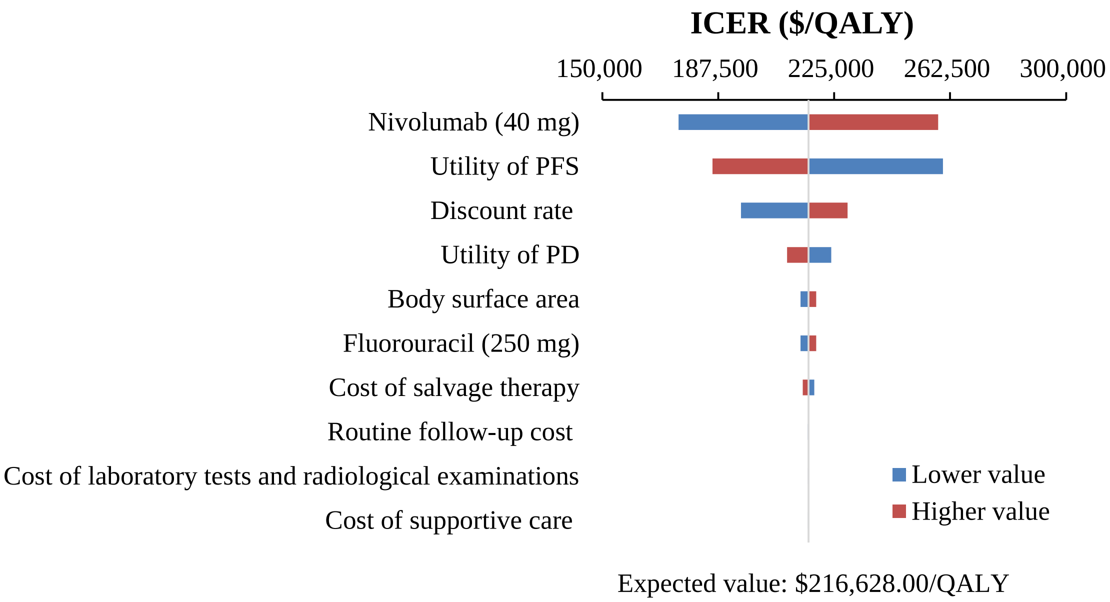

Supplementary Figure 14 | Tornado diagram of one-way sensitivity analysis of Nivolumab plus chemotherapy versus Chemotherapy in the treatment of PD-L1-positive advanced ESCC patients. ICER, incremental cost-effectiveness ratio; QALY, quality-adjusted life year; PFS, progression-free survival; PD, progressive disease.

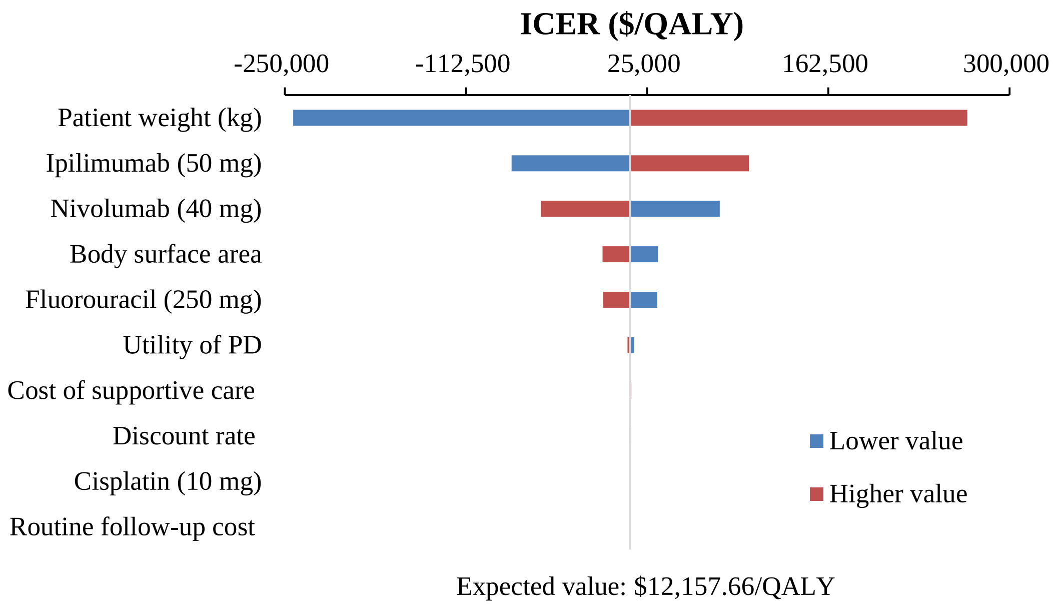

Supplementary Figure 15 | Tornado diagram of one-way sensitivity analysis of Nivolumab plus ipilimumab versus Nivolumab plus chemotherapy in the treatment of PD-L1-positive advanced ESCC patients. ICER, incremental cost-effectiveness ratio; QALY, quality-adjusted life year; PFS, progression-free survival; PD, progressive disease.

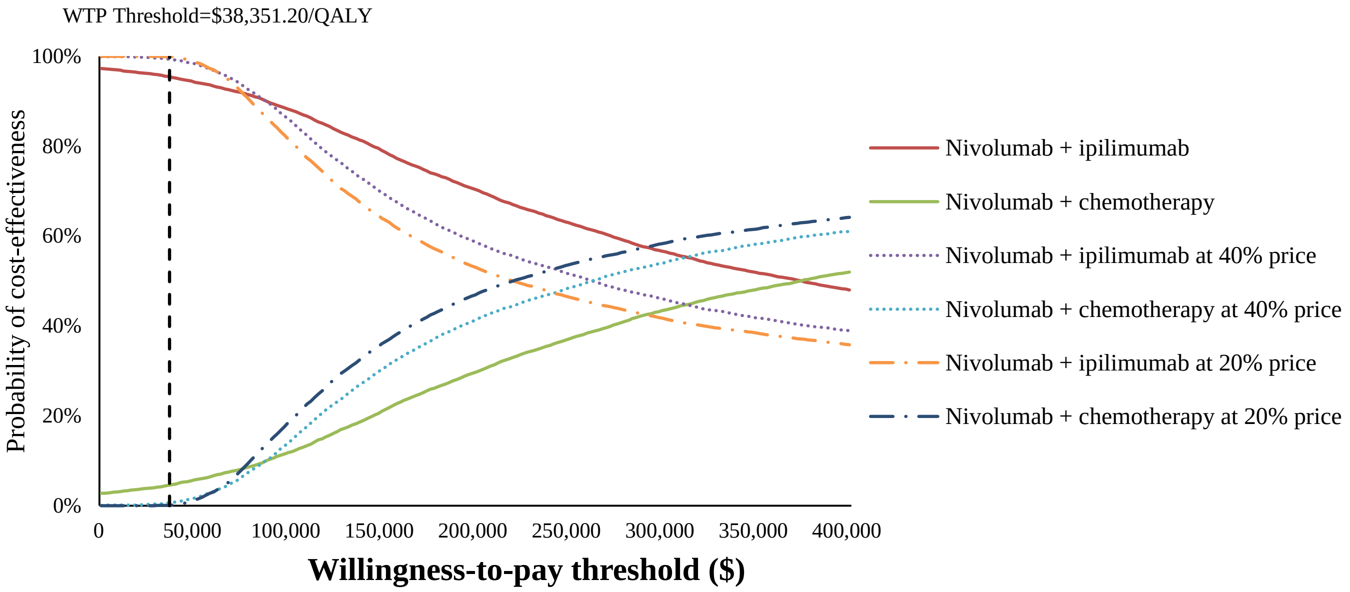

Supplementary Figure 16 | Cost-effectiveness acceptability curves of nivolumab plus chemotherapy versus nivolumab plus ipilimumab in the treatment of overall advanced ESCC patients from the Chinese healthcare perspective.

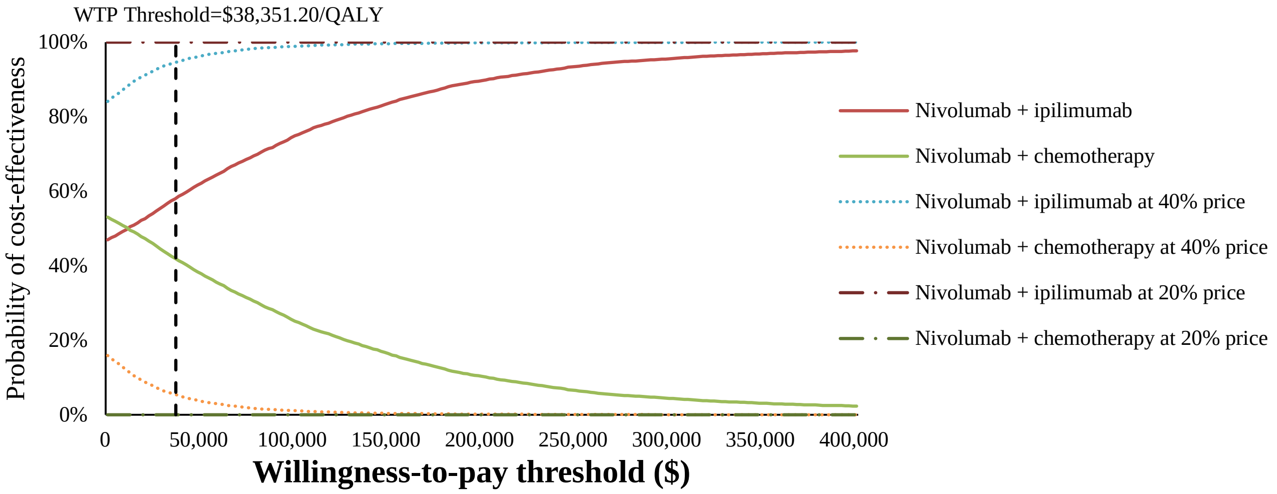

Supplementary Figure 17 | Cost-effectiveness acceptability curves of nivolumab plus chemotherapy versus nivolumab plus ipilimumab in the treatment of PD-L1-positive advanced ESCC patients from the Chinese healthcare perspective.
