# Supplementary material for: Cost-effectiveness analysis of nivolumab combination therapy in the first-line treatment for advanced esophageal squamous-cell carcinoma
Source: Front Oncol. 2022 Jul 22;12:899966. doi: 10.3389/fonc.2022.899966 (PMC9353037; doi:10.3389/fonc.2022.899966)
Supplement: Supplementary file 1 [file DataSheet_1.zip › Additional files/Supplementary Tables.pdf]

Supplementary Tables

Supplementary Table 1 | CHEERS Checklist 2022

Supplementary Table 2 | Summary of statistical goodness-of-fit of Kaplan-Meier curves in overall population

Supplementary Table 3 | Summary of statistical goodness-of-fit of Kaplan-Meier curves in Tumor-Cell PD-L1 Expression of ⩾1% patients

Supplementary Table 4 | Scenario analysis results (price reduction)

Supplementary Table 5 | Scenario analysis results (time horizon)

Supplementary Table 1 | CHEERS Checklist 2022

Section	Item No	Guidance for reporting	Reported
**Title**			
Title	1	Identify the study as an economic evaluation and specify the interventions being compared	Yes
**Abstract**			
Abstract	2	Provide a structured summary that highlights context, key methods, results, and alternative analyses	Yes
**Introduction**			
Background and objectives	3	Give the context for the study, the study question, and its practical relevance for decision making in policy or practice	Yes
**Methods**			
Health economic analysis plan	4	Indicate whether a health economic analysis plan was developed and where available	Yes
Study population	5	Describe characteristics of the study population (such as age range, demographics, socioeconomic, or clinical characteristics)	Yes
Setting and location	6	Provide relevant contextual information that may influence findings	Yes
Comparators	7	Describe the interventions or strategies being compared and why chosen	Yes
Perspective	8	State the perspective(s) adopted by the study and why chosen	Yes
Time horizon	9	State the time horizon for the study and why appropriate	Yes
Discount rate	10	Report the discount rate(s) and reason chosen	Yes
Selection of outcomes	11	Describe what outcomes were used as the measure(s) of benefit(s) and harm(s)	Yes
Measurement of outcomes	12	Describe how outcomes used to capture benefit(s) and harm(s) were measured	Yes
Valuation of outcomes	13	Describe the population and methods used to measure and value outcomes	Yes
Measurement and valuation of resources and costs	14	Describe how costs were valued	Yes
Currency, price date, and conversion	15	Report the dates of the estimated resource quantities and unit costs, plus the currency and year of conversion	Yes
Rationale and description of model	16	If modelling is used, describe in detail and why used. Report if the model is publicly available and where it can be accessed	Yes
Analytics and assumptions	17	Describe any methods for analysing or statistically transforming data, any extrapolation methods, and approaches for validating any model used	Yes
Characterising heterogeneity	18	Describe any methods used for estimating how the results of the study vary for subgroups	Yes
Characterising distributional effects	19	Describe how impacts are distributed across different individuals or adjustments made to reflect priority populations	Yes
Characterising uncertainty	20	Describe methods to characterise any sources of uncertainty in	Yes

		the analysis	
Approach to engagement with patients and others affected by the study	21	Describe any approaches to engage patients or service recipients, the general public, communities, or stakeholders (such as clinicians or payers) in the design of the study	Yes
**Results**			
Study parameters	22	Report all analytic inputs (such as values, ranges, references) including uncertainty or distributional assumptions	Yes
Summary of main results	23	Report the mean values for the main categories of costs and outcomes of interest and summarise them in the most appropriate overall measure	Yes
Effect of uncertainty	24	Describe how uncertainty about analytic judgments, inputs, or projections affect findings. Report the effect of choice of discount rate and time horizon, if applicable	Yes
Effect of engagement with patients and others affected by the study	25	Report on any difference patient/service recipient, general public, community, or stakeholder involvement made to the approach or findings of the study	Yes
**Discussion**			
Study findings, limitations, generalisability, and current knowledge	26	Report key findings, limitations, ethical or equity considerations not captured, and how these could affect patients, policy, or practice	Yes
Other relevant information Source of funding	27	Describe how the study was funded and any role of the funder in the identification, design, conduct, and reporting of the analysis	Yes
Conflicts of interest	28	Report authors conflicts of interest according to journal or International Committee of Medical Journal Editors requirements	Yes

Supplementary Table 2 | Summary of statistical goodness-of-fit of Kaplan-Meier curves in overall population

**Overall population**	**Exponential**	**Weibull**	**Log-logistic**	**Log-normal**	**Gompertz**
**Chemotherapy-PFS**					
parameter 1	0.128996	0.079119	1.694804	1.670192	0.120471
parameter 2	-	0.186124	-0.635573	-0.081726	0.010147
AIC	906.698000	891.655400	874.480400	870.814000*#	907.397500
BIC	910.478800	899.216800	882.041900	878.375500	914.958900
**Chemotherapy-OS**					
parameter 1	0.062475	0.025615	2.450230	2.408722	0.047178
parameter 2	-	0.266689	-0.620517	0.016033	0.025640
AIC	900.189300	875.601100	874.761300*#	905.908800	889.458300
BIC	903.970100	883.162500	882.322800	913.470300	897.019800
**Nivolumab + chemotherapy-PFS**					
parameter 1	0.088548	0.088336	1.908558	1.921162	0.108358
parameter 2	-	0.000860	-0.514040	0.057902	-0.020087
AIC	990.268500	992.268100	940.570800*#	946.132600	980.692900
BIC	994.039900	999.811000	948.113600	953.675500	988.235800
**Nivolumab + chemotherapy-OS**					
parameter 1	0.050684	0.018865	2.659128	2.617406	0.034479
parameter 2	-	0.268545	-0.598290	-0.034648	0.025629
AIC	886.604000	859.966100*	876.815600#	879.527200	865.222600
BIC	890.375400	867.509000	884.358400	887.070100	872.765400
**Nivolumab + ipilimumab-PFS**					
parameter 1	0.118615	0.183544	1.364702	1.447667	0.188410
parameter 2	-	-0.187945	-0.329654	0.197467	-0.060943
AIC	1134.555000	1116.450000	1048.817000#	1039.983000*	1079.617000
BIC	1138.339000	1124.017000	1056.385000	1047.551000	1087.185000
**Nivolumab + ipilimumab-OS**					
parameter 1	0.047934	0.042528	2.604049	2.563805	0.046947
parameter 2	-	0.038205	-0.305029	0.252755	0.001706
AIC	985.756900*	987.258400	991.208800#	993.279700	987.699000
BIC	989.540700	994.826000	998.776500	1000.847000	995.266600

**, best fitted model; #, best visual inspection; AIC, Akaike information criterion; BIC, Bayesian Information Criterion; PFS, progression-free survival; OS, overall survival.*

Supplementary Table 3 | Summary of statistical goodness-of-fit of Kaplan-Meier curves in Tumor-Cell PD-L1 Expression of ≥1% patients

Tumor-Cell PD-L1 Expression of ≥1%	Exponential	Weibull	Log-logistic	Log-normal	Gompertz
**Chemotherapy-PFS**					
parameter 1	0.1553414	0.0700908	1.558494	1.53971	0.103934
parameter 2	-	0.3143973	-0.6776225	-0.1628946	0.0757245
AIC	419.947900	401.269100	407.129200	398.106200*#	407.775900
BIC	423.004200	407.381600	413.241700	404.218700	413.888400
**Chemotherapy-OS**					
parameter 1	0.0615322	0.0253775	2.431702	2.423792	0.04994
parameter 2	-	0.2721876	-0.6274344	-0.0388095	0.0214299
AIC	431.013300	420.902800	411.081600*#	417.294400	429.970100
BIC	434.069600	427.015300	417.194100	423.406900	436.082600
**Nivolumab + chemotherapy-PFS**					
parameter 1	0.086203	0.070207	2.004654	1.996054	0.0925296
parameter 2	-	0.0767082	-0.4231975	0.1030589	-0.0095611
AIC	473.678500	474.584400	465.203100	461.741100*#	475.269600
BIC	476.741100	480.709600	471.328300	467.866300	481.394700
**Nivolumab + chemotherapy-OS**					
parameter 1	0.0394421	0.0154303	2.887272	2.845191	0.0250615
parameter 2	-	0.2568205	-0.4849153	0.0703652	0.0314207
AIC	433.869900	426.229600	433.582200	434.007000#	425.423000*
BIC	436.932500	432.354800	439.707400	440.132200	431.548200
**Nivolumab + ipilimumab-PFS**					
parameter 1	0.0858697	0.1852812	1.557834	1.657211	0.1712278
parameter 2	-	-0.3268428	-0.129916	0.3796985	-0.0802171
AIC	602.094100	577.467400	548.024300	542.875100*#	554.442900
BIC	605.156700	583.592600	554.149500	549.000300	560.568100
**Nivolumab + ipilimumab-OS**					
parameter 1	0.0381234	0.0395522	2.847587	2.809128	0.0373933
parameter 2	-	-0.0117112	-0.2071663	0.3819121	0.0014763
AIC	476.915600*	478.895900	482.794000	487.345900#	478.895700
BIC	479.978200	485.021100	488.919100	493.471100	485.020900

**, best fitted model; #, best visual inspection; AIC, Akaike information criterion; BIC, Bayesian Information Criterion; PFS, progression-free survival; OS, overall survival.*

Supplementary Table 4 | Scenario analysis results (price reduction)

**100% price of nivolumab and ipilimumab**						
	**Overall advanced ESCC patients**			**Advanced ESCC patients with PD-L1-positive status**		
Parameters	Chemotherapy	Nivolumab plus chemotherapy	Nivolumab plus ipilimumab	Chemotherapy	Nivolumab plus chemotherapy	Nivolumab plus ipilimumab
**Cost ($)**						
PFS state	7,991.72	89,759.94	73,645.06	6,645.37	98,330.56	98,712.79
PD state	5,047.23	1,628.02	2,452.71	5,605.15	2,286.57	2,795.46
Total Cost	14,499.25	92,848.26	77,558.07	13,710.82	102,077.43	102,968.54
**LYs**						
PFS state	0.62	0.91	0.68	0.51	1.02	1.04
PD state	0.45	0.45	0.69	0.84	1.13	1.53
Total LYs	1.08	1.36	1.38	1.35	2.15	2.57
**QALYs**						
PFS state	0.45	0.64	0.48	0.37	0.72	0.71
PD state	0.24	0.24	0.36	0.27	0.34	0.41
Total QALYs	0.70	0.88	0.84	0.65	1.05	1.13
**ICER ($/LYs)**	-	272,390.06	208,386.78	-	110,465.61	73,402.85
			-1,021,434.44*			2,141.84*
**ICER ($/QALY)**	-	415,163.81	430,704.11	-	216,628.00	185,483.94
			361,388.00*			12,157.66*
**80% price of nivolumab and ipilimumab**						
	**Overall advanced ESCC patients**			**Advanced ESCC patients with PD-L1-positive status**		
Parameters	Chemotherapy	Nivolumab plus chemotherapy	Nivolumab plus ipilimumab	Chemotherapy	Nivolumab plus chemotherapy	Nivolumab plus ipilimumab
**Cost ($)**						
PFS state	7,991.72	74,084.68	59,450.79	6,645.37	81,198.90	79,965.30

PD state	5,047.23	1,628.02	2,452.71	5,605.15	2,286.57	2,795.46
Total Cost	14,499.25	77,173.01	63,363.80	13,710.82	84,945.77	84,221.06
**LYs**						
PFS state	0.62	0.91	0.68	0.51	1.02	1.04
PD state	0.45	0.45	0.69	0.84	1.13	1.53
Total LYs	1.08	1.36	1.38	1.35	2.15	2.57
**QALYs**						
PFS state	0.45	0.64	0.48	0.37	0.72	0.71
PD state	0.24	0.24	0.36	0.27	0.34	0.41
Total QALYs	0.70	0.88	0.84	0.65	1.05	1.13
**ICER ($/LYs)**	-	217,893.08	161,479.82	-	89,049.62	57,985.49
			-922,499.56*			-1,741.87*
**ICER ($/QALY)**	-	332,102.14	333,754.47	-	174,630.27	146,525.33
			326,384.41*			-9,887.32*
**60% price of nivolumab and ipilimumab**						
	**Overall advanced ESCC patients**			**Advanced ESCC patients with PD-L1-positive status**		
Parameters	Chemotherapy	Nivolumab plus chemotherapy	Nivolumab plus ipilimumab	Chemotherapy	Nivolumab plus chemotherapy	Nivolumab plus ipilimumab
**Cost ($)**						
PFS state	7,991.72	58,409.43	45,256.52	6,645.37	64,067.24	61,217.82
PD state	5,047.23	1,628.02	2,452.71	5,605.15	2,286.57	2,795.46
Total Cost	14,499.25	61,497.75	49,169.53	13,710.82	67,814.11	65,473.58
**LYs**						
PFS state	0.62	0.91	0.68	0.51	1.02	1.04
PD state	0.45	0.45	0.69	0.84	1.13	1.53
Total LYs	1.08	1.36	1.38	1.35	2.15	2.57
**QALYs**						
PFS state	0.45	0.64	0.48	0.37	0.72	0.71

PD state	0.24	0.24	0.36	0.27	0.34	0.41
Total QALYs	0.70	0.88	0.84	0.65	1.05	1.13
**ICER ($/LYs)**	-	163,396.11	114,572.85	-	67,633.62	42,568.13
			-823,564.68*			-5,625.59*
**ICER ($/QALY)**	-	249,040.48	236,804.84	-	132,632.55	107,566.72
			291,380.81*			-31,932.30*
**40% price of nivolumab and ipilimumab**						
	**Overall advanced ESCC patients**			**Advanced ESCC patients with PD-L1-positive status**		
Parameters	Chemotherapy	Nivolumab plus chemotherapy	Nivolumab plus ipilimumab	Chemotherapy	Nivolumab plus chemotherapy	Nivolumab plus ipilimumab
**Cost ($)**						
PFS state	7,991.72	42,734.17	31,062.26	6,645.37	46,935.58	42,470.34
PD state	5,047.23	1,628.02	2,452.71	5,605.15	2,286.57	2,795.46
Total Cost	14,499.25	45,822.49	34,975.27	13,710.82	50,682.46	46,726.10
**LYs**						
PFS state	0.62	0.91	0.68	0.51	1.02	1.04
PD state	0.45	0.45	0.69	0.84	1.13	1.53
Total LYs	1.08	1.36	1.38	1.35	2.15	2.57
**QALYs**						
PFS state	0.45	0.64	0.48	0.37	0.72	0.71
PD state	0.24	0.24	0.36	0.27	0.34	0.41
Total QALYs	0.70	0.88	0.84	0.65	1.05	1.13
**ICER ($/LYs)**	-	108,899.13	67,665.89	-	46,217.62	27,150.76
			-724,629.80*			-9,509.30*
**ICER ($/QALY)**	-	165,978.81	139,855.20	-	90,634.82	68,608.11
			256,377.22*			-53,977.29*

20% price of nivolumab and ipilimumab						
	**Overall advanced ESCC patients**			**Advanced ESCC patients with PD-L1-positive status**		
Parameters	Chemotherapy	Nivolumab plus chemotherapy	Nivolumab plus ipilimumab	Chemotherapy	Nivolumab plus chemotherapy	Nivolumab plus ipilimumab
**Cost ($)**						
PFS state	7,991.72	27,058.91	16,867.99	6,645.37	29,803.93	23,722.86
PD state	5,047.23	1,628.02	2,452.71	5,605.15	2,286.57	2,795.46
Total Cost	14,499.25	30,147.24	20,781.00	13,710.82	33,550.80	27,978.62
**LYs**						
PFS state	0.62	0.91	0.68	0.51	1.02	1.04
PD state	0.45	0.45	0.69	0.84	1.13	1.53
Total LYs	1.08	1.36	1.38	1.35	2.15	2.57
**QALYs**						
PFS state	0.45	0.64	0.48	0.37	0.72	0.71
PD state	0.24	0.24	0.36	0.27	0.34	0.41
Total QALYs	0.70	0.88	0.84	0.65	1.05	1.13
**ICER ($/LYs)**	-	54,402.16	20,758.92	-	24,801.62	11,733.40
			-625,694.93*			-13,393.01*
**ICER ($/QALY)**	-	82,917.15	42,905.57	-	48,637.09	29,649.50
			221,373.62*			-76,022.27*

**, nivolumab plus chemotherapy versus Nivolumab plus ipilimumab; ESCC, esophageal squamous-cell carcinoma; PFS, progression-free survival; OS, overall survival; LYs, lifeyears; QALYs, Quality-adjusted life years; ICER, Incremental cost-effectiveness ratio.*

Supplementary Table 5 | Scenario analysis results (time horizon)

Time Horizon = 2 years						
	Overall advanced ESCC patients			Advanced ESCC patients with PD-L1-positive status		
Parameters	Chemotherapy	Nivolumab plus chemotherapy	Nivolumab plus ipilimumab	Chemotherapy	Nivolumab plus chemotherapy	Nivolumab plus ipilimumab
**Cost ($)**						
PFS state	7,466.75	87,633.96	71,872.50	6,430.77	95,798.77	94,972.88
PD state	3,192.03	997.18	1,260.34	3,679.59	1,074.42	1,085.30
Total Cost	12,119.09	90,091.44	74,593.14	11,570.66	98,333.49	97,518.48
**LYs**						
PFS state	0.58	0.72	0.52	0.49	0.79	0.69
PD state	0.27	0.26	0.33	0.31	0.28	0.28
Total LYs	0.84	0.98	0.84	0.80	1.07	0.97
**QALYs**						
PFS state	0.42	0.52	0.38	0.36	0.57	0.50
PD state	0.15	0.15	0.19	0.18	0.16	0.16
Total QALYs	0.58	0.67	0.56	0.54	0.73	0.66
**ICER ($/LYs)**	-	597,223.47	-26,851,216.00	-	330,783.90	513,933.74
			116,629.65*			8,573.72*
**ICER ($/QALY)**	-	822,991.92	-5,731,696.53	-	454,520.33	704,825.98
			146,705.47*			11,820.88*
**Time Horizon = 5 years**						
	Overall advanced ESCC patients			Advanced ESCC patients with PD-L1-positive status		
Parameters	Chemotherapy	Nivolumab plus chemotherapy	Nivolumab plus ipilimumab	Chemotherapy	Nivolumab plus chemotherapy	Nivolumab plus ipilimumab
**Cost ($)**						
PFS state	7,939.22	89,176.63	73,078.80	6,632.30	97,787.92	97,556.32

PD state	4,533.11	1,462.39	2,025.98	5,100.43	1,901.41	2,080.03
Total Cost	13,932.63	92,099.32	76,565.08	13,193.03	101,149.63	101,096.65
**LYs**						
PFS state	0.62	0.85	0.62	0.51	0.96	0.92
PD state	0.40	0.39	0.55	0.44	0.52	0.57
Total LYs	1.01	1.24	1.17	0.95	1.48	1.49
**QALYs**						
PFS state	0.45	0.61	0.45	0.37	0.68	0.65
PD state	0.22	0.22	0.30	0.25	0.28	0.31
Total QALYs	0.67	0.83	0.75	0.62	0.97	0.96
**ICER ($/LYs)**	-	336,422.58	397,285.13	-	167,855.99	165,303.73
			207,967.25*			-6,817.83*
**ICER ($/QALY)**	-	491,242.12	792,078.77	-	254,620.47	262,318.75
			194,064.02*			5,123.44*
**Time Horizon = 8 years**						
	**Overall advanced ESCC patients**			**Advanced ESCC patients with PD-L1-positive status**		
Parameters	Chemotherapy	Nivolumab plus chemotherapy	Nivolumab plus ipilimumab	Chemotherapy	Nivolumab plus chemotherapy	Nivolumab plus ipilimumab
**Cost ($)**						
PFS state	7,984.63	89,614.51	73,493.09	6,644.01	98,220.52	98,421.36
PD state	4,921.86	1,588.68	2,334.23	5,484.55	2,193.34	2,585.91
Total Cost	14,366.79	92,663.49	77,287.62	13,588.86	101,874.15	102,467.57
**LYs**						
PFS state	0.62	0.90	0.67	0.51	1.01	1.01
PD state	0.44	0.43	0.65	0.49	0.61	0.74
Total LYs	1.06	1.33	1.31	1.00	1.62	1.74
**QALYs**						
PFS state	0.45	0.63	0.47	0.37	0.71	0.70

PD state	0.24	0.24	0.35	0.27	0.32	0.38
Total QALYs	0.69	0.87	0.82	0.64	1.03	1.08
**ICER ($/LYs)**	-	287,653.40	245,522.86	-	141,822.26	119,007.25
			965,923.30*			4,773.03*
**ICER ($/QALY)**	-	432,212.41	496,428.19	-	224,176.78	202,164.18
			282,612.46*			12,952.15*
**Time Horizon = 10 years**						
	**Overall advanced ESCC patients**			**Advanced ESCC patients with PD-L1-positive status**		
Parameters	Chemotherapy	Nivolumab plus chemotherapy	Nivolumab plus ipilimumab	Chemotherapy	Nivolumab plus chemotherapy	Nivolumab plus ipilimumab
**Cost ($)**						
PFS state	7,991.72	89,759.94	73,645.06	6,645.37	98,330.56	98,712.79
PD state	5,047.23	1,628.02	2,452.71	5,605.15	2,286.57	2,795.46
Total Cost	14,499.25	92,848.26	77,558.07	13,710.82	102,077.43	102,968.54
**LYs**						
PFS state	0.62	0.91	0.68	0.51	1.02	1.04
PD state	0.45	0.45	0.69	0.84	1.13	1.53
Total LYs	1.08	1.36	1.38	1.35	2.15	2.57
**QALYs**						
PFS state	0.45	0.64	0.48	0.37	0.72	0.71
PD state	0.24	0.24	0.36	0.27	0.34	0.41
Total QALYs	0.70	0.88	0.84	0.65	1.05	1.13
**ICER ($/LYs)**	-	272,390.06	208,386.78	-	110,465.61	73,402.85
			-1,021,434.44*			2,141.84*
**ICER ($/QALY)**	-	415,163.81	430,704.11	-	216,628.00	185,483.94
			361,388.00*			12,157.66*

Time Horizon = 15 years						
	Overall advanced ESCC patients			Advanced ESCC patients with PD-L1-positive status		
Parameters	Chemotherapy	Nivolumab plus chemotherapy	Nivolumab plus ipilimumab	Chemotherapy	Nivolumab plus chemotherapy	Nivolumab plus ipilimumab
**Cost ($)**						
PFS state	7,996.19	89,945.47	73,856.25	6,646.05	98,434.91	99,073.07
PD state	5,200.97	1,675.07	2,622.35	5,750.76	2,392.01	3,104.03
Total Cost	14,657.46	93,080.84	77,938.90	13,857.11	102,287.21	103,637.40
**LYs**						
PFS state	0.62	0.94	0.71	0.51	1.03	1.09
PD state	0.48	0.47	0.77	0.52	0.70	0.96
Total LYs	1.10	1.41	1.48	1.03	1.73	2.05
**QALYs**						
PFS state	0.45	0.65	0.49	0.37	0.72	0.73
PD state	0.25	0.25	0.39	0.28	0.35	0.46
Total QALYs	0.70	0.90	0.88	0.65	1.08	1.19
**ICER ($/LYs)**	-	252,283.11	164,671.77	-	127,210.94	88,806.71
			-206,198.75*			4,275.22*
**ICER ($/QALY)**	-	395,156.77	360,032.05	-	209,386.11	166,206.53
			667,184.05*			11,457.54*

**, nivolumab plus chemotherapy versus Nivolumab plus ipilimumab; ESCC, esophageal squamous-cell carcinoma; PFS, progression-free survival; OS, overall survival; LYs, lifeyears; QALYs, Quality-adjusted life years; ICER, Incremental cost-effectiveness ratio.*
